# Antimicrobial and antitumor properties of anuran peptide temporin-SHf induce apoptosis in A549 lung cancer cells

**DOI:** 10.1007/s00726-023-03373-3

**Published:** 2024-02-06

**Authors:** Anet Antony, Anupama Kizhakke Purayil, Shilpa Olakkaran, Shweta Dhannura, Shamasoddin Shekh, Konkallu Hanumae Gowd, Hunasanahally Puttaswamygowda Gurushankara

**Affiliations:** 1 Department of Zoology, School of Biological Sciences, Central University of Kerala, Tejaswini Hills, Periya, Kasaragod, 671 320 India; 2 Department of Chemistry, School of Chemical Sciences, Central University of Karnataka, Kalaburagi, Karnataka 585 367 India; 3 Department of Zoology, University of Calicut, Malappuram, Kerala 673 635 India; 4 Department of Molecular Biology, Kannur University, Dr. Janakiammal Campus, Thalasserry, Palayad, Kerala 670 661 India

**Keywords:** Temporin-SHf, Cytotoxicity, A549 cells, Angiogenesis, Apoptosis, Anticancer peptides

## Abstract

Temporin-SHf is a linear, ultra-short, hydrophobic, α-helix, and phe-rich cationic antimicrobial peptide. The antitumor activities and mechanism of temporin-SHf-induced cancer cell death are unknown. The temporin-SHf was synthesized by solid-phase Fmoc chemistry and antimicrobial and antitumor activities were investigated. Temporin-SHf was microbiocidal, non-hemolytic, and cytotoxic to human cancer cells but not to non-tumorigenic cells. It affected the cancer cells' lysosomal integrity and caused cell membrane damage. The temporin-SHf inhibited A549 cancer cell proliferation and migration. It is anti-angiogenic and causes cancer cell death through apoptosis. The molecular mechanism of action of temporin-SHf confirmed that it kills cancer cells by triggering caspase-dependent apoptosis through an intrinsic mitochondrial pathway. Owing to its short length and broad spectrum of antitumor activity, temporin-SHf is a promising candidate for developing a new class of anticancer drugs.

**Supplementary Information:**

The online version contains supplementary material available at 10.1007/s00726-023-03373-3.

## Introduction

Cancer is a global public health problem. Despite many advances in cancer therapy, it remains the second leading cause of death worldwide (Siegel et al. 2023). Traditional chemotherapeutics are non-specific for cancer cells, and the onset of drug resistance is the most common cause of tumor recurrence and consequent undesirable side effects for the patients (Al-Mugotir et al. 2021; Tiek and Cheng 2022). Advanced therapies, such as endocrine-based ones, develop secondary malignancies (Patel et al. 2023). Angiogenic inhibitors have shown adverse side effects such as hypertension, teratogenicity, and proteinuria (Jászai and Schmidt 2019). The limited penetration of anticancer drugs through tumor tissue is the potential cause of clinical resistance of solid tumors to chemotherapy (Tannock et al. 2002). Thus, new, safe, and efficient therapeutics that can kill cancer cells selectively and a different mechanism of action need to be developed.

Natural drug discovery represents an area of research with vast potential. Naturally occurring peptides have the potential to provide the most promising alternative to currently available antibiotics and cancer drugs (Luong et al. 2020). Antimicrobial peptides (AMPs), also called host defense peptides, have a great interest as potential peptide-based drug discovery for human health (Kardani and Bolhassani 2021). They are small bioactive proteins naturally produced by almost all living organisms, such as microorganisms, insects, plants, birds, fishes, amphibians, mammals, and humans. AMPs represent the innate defense against fungi, viruses, and bacteria (Magana et al. 2020). AMPs isolated from various organisms have shown their microbiocidal and cytolytic properties (Wang et al. 2012). These natural peptide-based antimicrobial agents are available today to treat infections, wound healing, and metabolic syndrome (Mookherjee et al. 2020). About 3,457 AMPs have been discovered, characterized, and annotated in the AMP database, considering that amphibian skin alone is a reservoir of more than 1148 different AMPs. The main functions of antimicrobial peptides include antibacterial peptides (84.50%), followed by antifungal peptides (36.56%), and anticancer (antitumor) peptides (7.69%) (APD3 2023).

Amphibians, especially anurans (frogs and toads), have a rich collection of bioactive peptides that are produced from granular (poison) glands and secreted towards the skin surface following constant exposure to microbial stimulations (Patocka et al. 2019). They are essential in protecting them from invasion by various microorganisms, including the chytrid fungus *Batrachochytrium dendrobatids* implicated in population decline in certain amphibian species (Rollins-Smith 2023). The amphibian skin secretions are extraordinarily diverse and have raised the interest of biochemists as a "natural pharmacy." In many ancient cultures, amphibian skin and its secretions are believed to possess medicinal value and have long been used in ethnic/traditional folklore medicines. Anuran skin is burned to ashes and used in medicinal formulae and concoctions for many illnesses (Wang et al. 2020). Amphibians are considered a storehouse of pharmaceutical products. Some compounds are extracted and used as painkillers to treat trauma such as burns and heart attacks (Conlon 2011). The structure of AMPs is generally 8–50 amino acid residues long, linear, cyclic, and simple-structured, the majority being hydrophobic, cationic, and possessing an amphipathic α-helix in nature (Conlon et al. 2019). These properties of AMPs are responsible for executing their biological activity with practically inactive hemolysis (Patocka et al. 2019). The AMPs derived from amphibian skin secretions can also kill cancer cells (Wang et al. 2012; Anet et al. 2022); when administered locally to solid tumors, hematopoietic malignancies inhibited metastasis without producing detectable side effects (Cerón et al. 2010). AMPs' high targetability and penetrability and low immunogenicity and molecular weight favour developing anticancer drugs (Wang et al. 2013). The AMPs inhibiting tumor cell proliferation are similar to the antimicrobial mechanism, such as membrane lysis: plasma membrane disruption, or non-membranolytic cytotoxicity (Tornesello et al. 2020). The anionic compositions of cancer cell membranes may be perfect targets for AMPs with additional tumoricidal properties, called cationic anticancer peptides (ACPs) (Hoskin and Ramamoorthy 2008; Oelkrug et al. 2015). These ACPs have selectively killed the tumor cells or MDR bacteria with lower side effects by distinct mechanisms (Riedl et al. 2011). Some AMPs induce the release of tumor antigens and potent damage-associated molecular patterns by causing alterations in the intracellular organelles of cancer cells (Sveinbjørnsson et al. 2017). ACPs were isolated from amphibian skin secretions targeting different cancers, which can inhibit tumor cell proliferation, migration, and angiogenesis (Pan et al. 2020). The clinical applications of prospective ACPs are promising alternatives to conventional chemotherapy, suggesting a candidate for developing unique peptide-based therapeutics, alone or in combination with other small molecules, in oncology.

Temporins are a family of shortest natural peptides (8–17 amino acids) (average 13.67), which do not contain cysteine (no disulfide bonds). These are linear α-helical peptides containing a C-terminally α-amidated residue with a net charge between 0 to + 4 (average + 1.02) and hydrophobic content in 46 to 76% (average 63.06%). Temporins were first isolated from the skin secretions of the Asian frog *Rana erythraea* (Yasuhara et al. 1985) and the European frog *Rana esculenta* (Simmaco et al. 1990)*.* They were also identified in the skin of the European red frog *R. temporaria* (Simmaco et al. 1996). The term "temporin" was first used by Simmaco and coworkers to describe a family of ten structurally related peptides identified based on the consensus sequences (FLPLIASLLSKLL.NH_2_) has much phenylalanine (Phe) residue, anchoring by direct magnetic dipole–dipole interactions between aromatic rings of Phe and cell membranes anionic phosphatidylglycerol (PG) (Wang et al. 2005). Temporins are the smallest AMPs found in nature. They can perturb the integrity of target cell membranes (i.e., through the formation of pore-like openings) and show their antimicrobial and anticancer properties without causing cytolysis or haemolysis in normal cells (Rinaldi et al. 2002; Wang et al. 2012; Mishra et al. 2018). Temporin-SHf (FFFLSRIFa) is a linear, ultra-short, hydrophobic,α-helix, Phe-rich peptide (50%) isolated from the skin secretions of *Pelophylax saharica,* demonstrating a broad spectrum of antimicrobial activities particularly against multidrug-resistant bacterial strains with lack of hemolysis (Abbassi et al. 2010; André et al. 2015). The four Phe residues in this wild frog peptide are reminiscent of human cathelicidin LL-37 features and are remarkably conserved in temporin-SHf, making it a minimal LL-37-like peptide (Wang et al. 2019). With the disulfide engineering strategy, hydrophobic residues were mutated with hydrophobic cysteine disulfide to yield designed temporin‐SHf and have a chemical nature to that of native peptide, showing improved stability and antimicrobial activity (Dolle et al. 2019). Temporin‐SHf is a good model for peptidomimetic studies and for developing viable temporin‐SHf‐based antimicrobial/anticancer drugs. However, no studies regarding the antitumor activity of temporin-SHf have been reported to date. Further, the molecular basis for the selective targeting of cancer cells and the mechanism of cell killing by host defense peptides remains unknown. In this background, the present study analyzed the antimicrobial and anticancer activities of temporin-SHf and its molecular mechanism of action against cancer cells.

## Results and discussion

### Characterization of temporin-SHf

**Fig. 1:**
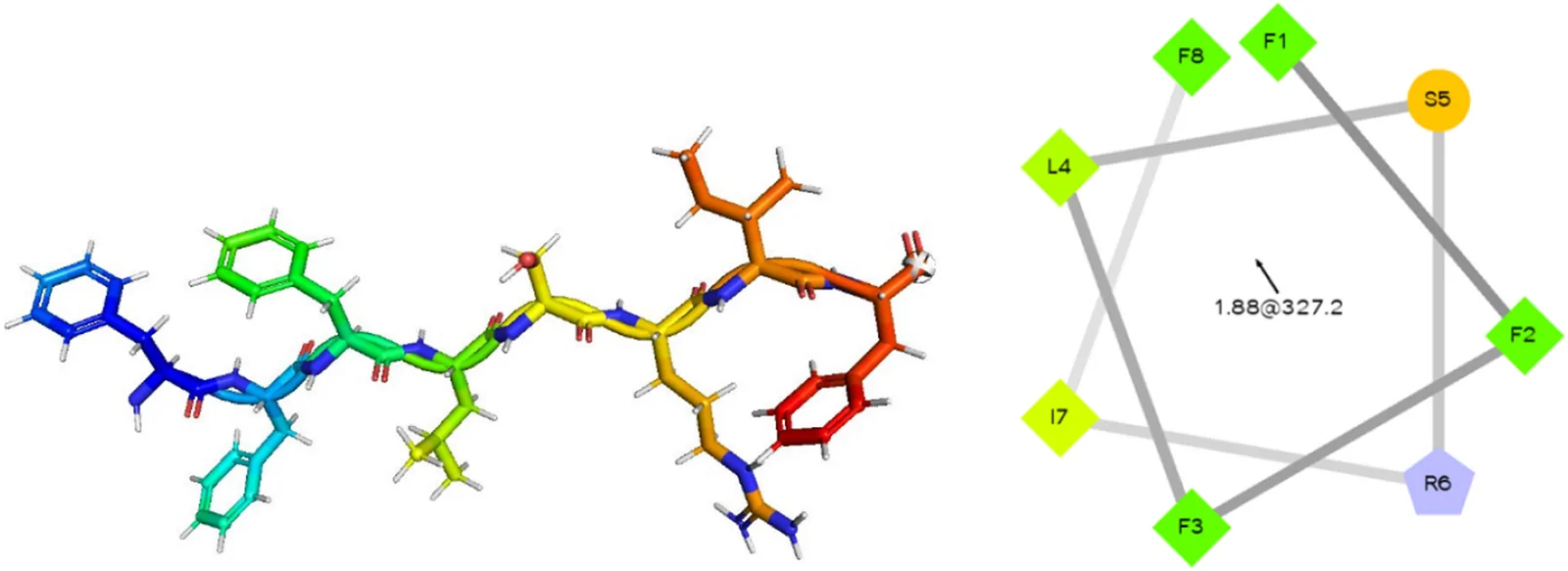
Secondary structure of temporin-SHf. Prediction of amphiphilicity of the temporin-SHf and distribution of hydrophobic and hydrophilic amino acid residues on the helical wheel plot. The hydrophilic residue is the circle, hydrophobic residues as diamonds, and potentially positively charged as pentagons. Hydrophobicity is color-coded: the most hydrophobic residue is green, and the amount of green decreases proportionally to the hydrophobicity, with zero hydrophobicity coded as yellow. The potentially charged residues are light blue

Technology such as solid-phase peptide synthesis (SPSS) allows a sufficient amount of newly isolated peptides for detailed characterization and analysis of their biological activities (Merrifield et al. 1994). The highly hydrophobic temporin-SHf sequence (FFFLSRIF*) was synthesized using SPPS Fmoc chemistry. The observed mass of temporin-SHf is consistent with the previous report by Abbassi et al. (2010) on the isolation and characterization of temporin-SHf from the skin secretion of *Pelophylax saharica*. As reported in our earlier publication (Dolle et al. 2019), ESI–MS/MS of [M + 2H]^2+^ ion of temporin-SHf confirms the fragment series of b and y ions observed in the fragmentation spectrum of temporin-SHf. The fragments at *m*/*z* 295, 442, 798, and 911 correspond to *b2, b3, b6,* and *b7* ions. The fragments at *m*/*z* 165, 63,782, and 928 correspond to *y1, y5, y6,* and *y7* ions, respectively. These observed fragment ions are as per the sequence of temporin-SHf. The purity of the temporin-SHf was more than 99% (Fig. S1) and was used for functional studies to evaluate the biological activity of the peptide. The temporin-SHf has75% hydrophobicity (Table 1), contributing to the peptides' affinity towards the cell membrane. The hydrophobicity of peptides is correlated with their antimicrobial, hemolytic, and antitumor activities (Manrique-Moreno et al. 2021). Helix wheel analysis showed that the temporin-SHf had an α-helical structure and high amphiphilicity. All the hydrophobic amino acids are on one side of the helix. In contrast, polar or hydrophilic amino acids were on the other side, forming the hydrophobic helix face (Fig. 1), and this structure enhances membrane disruption (Lin et al. 2022).

**Table 1:** Physicochemical properties of anuran skin peptide temporin-SHf

Peptide name	Temporin-SHf
Chemical formula	C_57_H_77_N_11_O_10_
Peptide sequence	FFFLSRIF-Am
No. of Amino acids	8
Secondary structure prediction	α-helix/linear
Net charge	+ 1
Mass (in Da)	1076.31
Isoelectric point (pI)	9.75
Hydrophobicity(%)	75
Grand average of hydropathicity (GRAVY)	1.775

### 3D structure of temporin-SHf

**Fig. 2:**
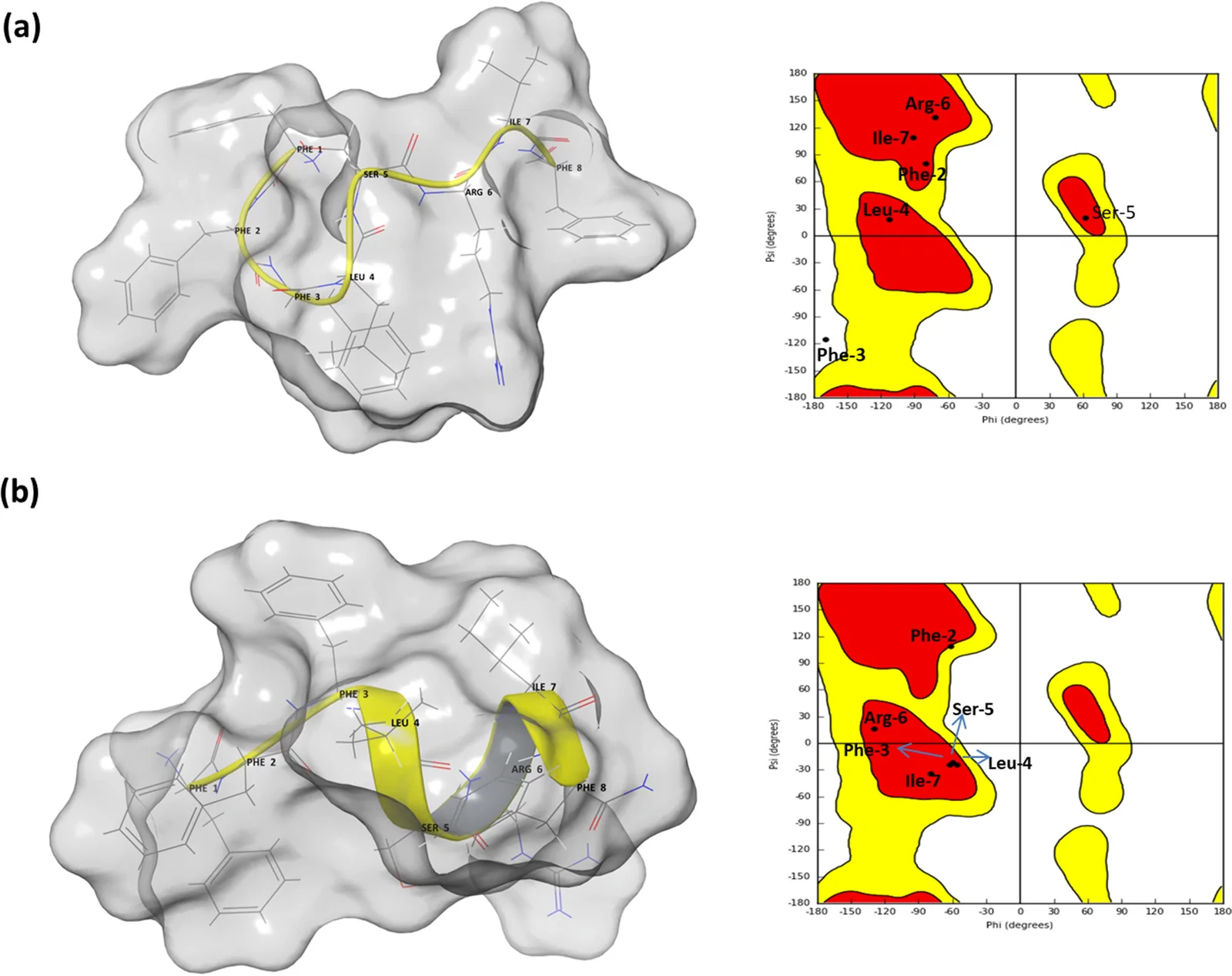
The 3D modeled structure of Temporin-SHf. **a** In water, **b** In micelles. The temporin-L structure in water and in SDS micelles was used as templates. The corresponding Ramachandran maps are also indicated

The 3D-structure of temporin-SHF has been deduced using homology modeling on the glide platform of Schrodinger software. The two temporin-related peptides namely temporin-L and temporin-B are available in PDB (Fig. S2), and the temporin-SHf closely aligned with that of the temporin-L. Thus, the temporin-L structure is used as a template for homology studies. The structure of the temporin-SHf in solution and the presence of micelles were deduced as the corresponding templates of temporin-L available in the database. The details of modeling of 3D structure of temporin are described in the method section. After the modeling, the peptide structures were minimized using a protein preparation wizard and subjected to Ramachandran map analysis. In solution and the presence of SDS micelles, that the temporin-SHf has a random coil structure in solution and adopts a partial helical fold in SDS micelles. These structural changes were also evident from the Ramachandran map (Fig. 2). In the SDS micelles, the temporin-SHf adopts a helical structure from residue Phe-3 to Phe-8. These results also align with the experimental structural analysis of temporin-SHf using NMR spectroscopy (Abbassi et al. 2010). Temporin-SHf has a disordered structure in water and adopts helical conformation from the segment Phe-3 to Phe-8 residues in micelles. The similarity in structures was evident between NMR structure and homology modeled structure; (1) hydrogen bonding between Phe-8-NH– Leu-4-CO, and Ile-7-NH– Phe-3-CO. In the modeled structure of temporin-SHf in SDS micelles, the hydrogen bond distance between Phe-8-NH and Leu-4-CO is 2.15 Å, Ile-7-NH to Leu-4-CO is 2.27 Å, and Arg-6-NH to Phe-3-CO is 2.24 Å (Fig. S3a). Further Phe-2 and Phe-1 tended to aromatic Phe-Phe T-shaped interaction that may be leading to the distorted structure towards the N-terminus of temporin-SHf (Fig. S3b). These observations indicate that the temporin-SHf adopts alpha-helical conformation.

### Antibacterial and antifungal activities of temporin-SHf

The outer membrane (OM) of Gram-negative bacteria has lipopolysaccharides (LPS), and the inner membrane (IM) contains phosphatidylglycerols (PGs) and phosphatidylethanolamine (PEs) (Epand 2019). Typically, *E.coli* is a model strain for antibacterial assay. Many amphibian peptides are active against Gram-negative bacteria that can penetrate the OM and exert their damaging effects on IM (Zasloff 2002). Gram-positive bacteria do not have an OM but possess a cell wall (Epand 2019). *Staphylococcus aureus* is a model strain, which displays increasing resistance to currently available antibiotics (Di Grazia et al. 2014). Temporin-SHf is microbiocidal to Gram-negative and Gram-positive bacteria; but has high potency against *E. coli* Gram-negative bacteria (Table 2). The temporins are generally active against Gram-positive bacteria such as methicillin-resistant *Staphylococcus aureus* (MRSA), *S. epidermidis, B. subtilis,* and *E. faecium,* with MIC values ranging from 1 to > 100 µM (Mishra et al. 2018). Other amphibian peptides, such as the brevinin-2 from *Rana esculenta* and *R. ornativentris* showed high potency against *E. coli* (MIC < 10 µM) but were also active against *S. aureus* (Wang et al. 1998). Esculentin-2 shows broad-spectrum antimicrobial activity with high potency against *E. coli* and *S. aureus* (MIC < 10 µM) (Simmaco et al. 1994). The MIC values against *E. coli* range from 2 µM (ranatuerin-2Cb) to 30 µM (ranatuerin-2ARa) and against *S. aureus* from 2 µM (ranatuerin-2B) to > 200 µM (ranatuerin-2ARa) (Goraya et al. 1998). The temporin L from *R. temporaria* bears a net positive charge of + 3 and is active against clinically relevant Gram-negative strains such as *E. coli* and *P. aeruginosa* (Rinaldi et al. 2002). Peptide antimicrobial activity depends on its hydrophobicity, cationic, and amphipathic α-helical conformation (Tossi et al. 2000). The balance between hydrophobicity and charge is crucial for antimicrobial activity, membrane permeabilization, and cytotoxicity (Liu et al. 2019). Temporin-SHf has a high hydrophobic (75%) and net charge of + 1. The amino acid Phe is more abundant; it folds into a non-amphipathic hydrophobic α-helix (Table 1), a feature that might have been responsible for its spectrum of antimicrobial activity (Table 2).

**Table 2:** Antibacterial activity of temporin-SHf

Sl. No	Bacterial strains	MIC (μM)
	Gram‐negative bacteria	
1.	*Escherichia coli* (ATCC 25922)	24.74 ± 0.968
2.	*Pseudomonas aeruginosa* (ATCC 15442)	108.32 ± 1.758
3.	*Klebsiella pneumonia* (ATCC 13883)	42.56 ± 1.455
4.	*Aeromonas hydrophila* (ATCC 7966)	167.35 ± 2.127
	Gram‐positive bacteria	
5.	*Staphylococcus aureus* (MTCC 9542)	34.07 ± 1.632
6.	*Bacillus subtilis* (ATCC 6051)	39.27 ± 1.359

These concentrations represent the minimum dose required to kill the entire bacteria

*MIC* minimum inhibitory concentration

**Fig. 3:**
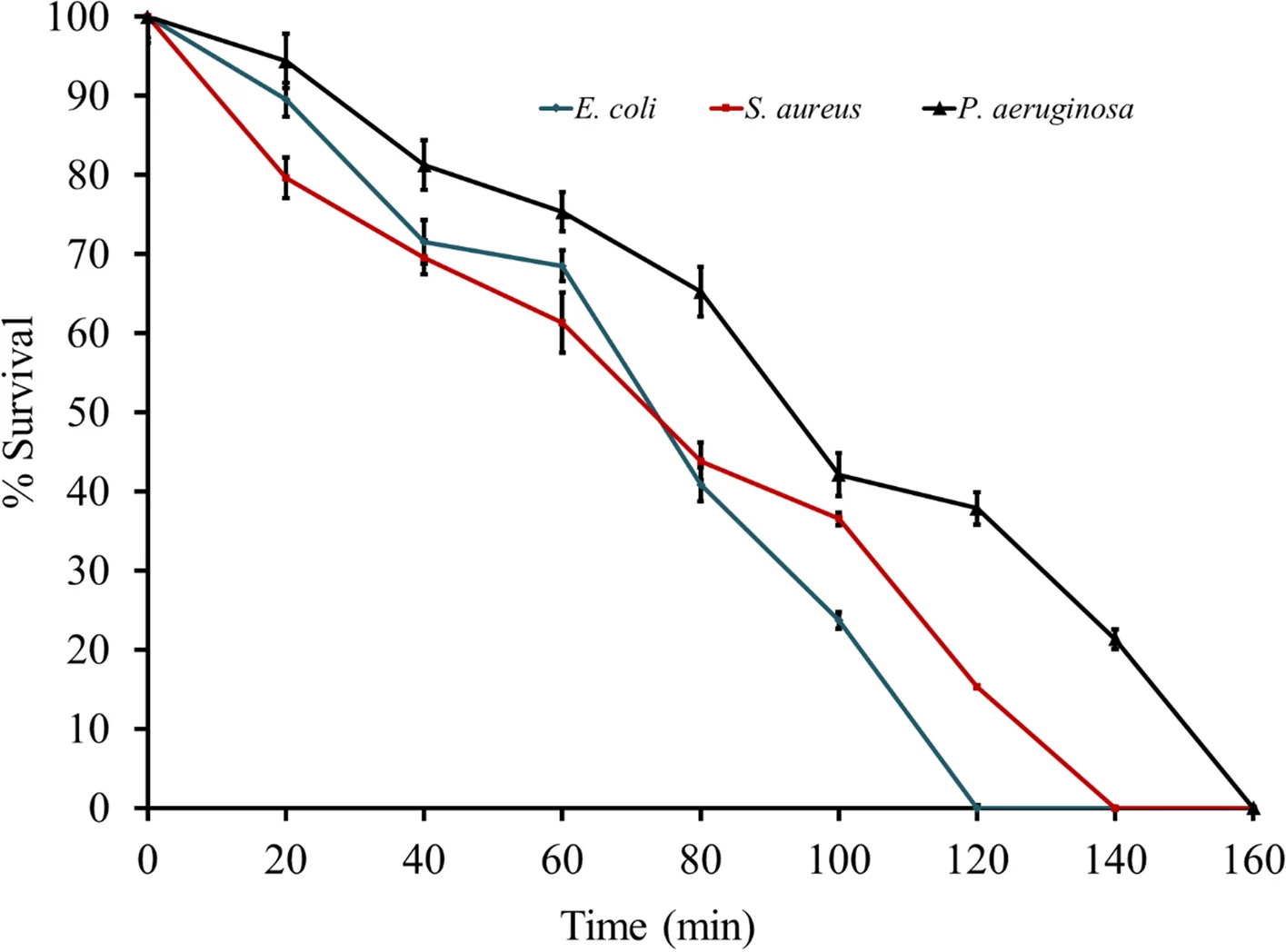
Killing kinetics of bacteria by temporin-SHf. *E. coli, S. aureus,* and *P.aeuginosa* were treated with the MIC concentration of temporin-SHf

Temporin-SHf treated with the different periods from 1 to 3 h duration showed a similar curve shape; it takes 120–160 min to kill *E.coli*, *S. aureus*, and *P.aeuginosa* (Fig. 3), which indicated that temporin-SHf exerted its antibacterial activity rapidly and non-time-dependently. Temporins produce bacterial cell death by forming transmembrane pores of the cell membrane into peptide-coated vesicles; perturbation of bilayer organization occurs through pore-like openings (Mangoni et al. 2000). It can be concentration-dependent; a pore may form on bacterial membranes at the MIC at micromolar or µg/mL level (Wang 2020). There seemed to be a concentration threshold for the antimicrobial activity of the temporin-SHf. The mechanism of action by which hydrophobic peptides kill microbes is insertion into the hydrophobic core of the cell membrane, interaction with anionic heads and hydrocarbon tails of microbes' phospholipids, binding to DNA or altering enzyme activities (Simonetti et al. 2008). AMPs are also more advantageous than conventional antibiotics to target intracellular components. One of the mechanisms of bactericidal activity is the direct disruption of bacterial membrane electric potentials, which results in less likelihood for cross-resistance development (Kamysz et al. 2007). The membrane-disturbing mechanism of AMPs makes it difficult for the microbes to develop drug resistance because the composition of the microbial membrane is relatively stable and difficult to mutate (Mangoni 2006). As peptides are less susceptible to developing microbial resistance, they have attracted considerable interest as a possible new generation of antimicrobials, especially for AMR pathogens. Based on this mode of action, the temporin-SHf is unlikely to cause rapid emergence of resistance, because it would require significant alteration of membrane composition, which is highly unlikely (Peschel and Sahl 2006; Yeaman and Yount 2003). Moreover, temporin-SHf differs from conventional antibiotics that might kill microbes through interaction with specific and discrete molecular targets (Chan et al. 2006). These exciting features make AMPs attractive therapeutic agents for treating infections caused by MDR pathogens.

**Fig. 4:**
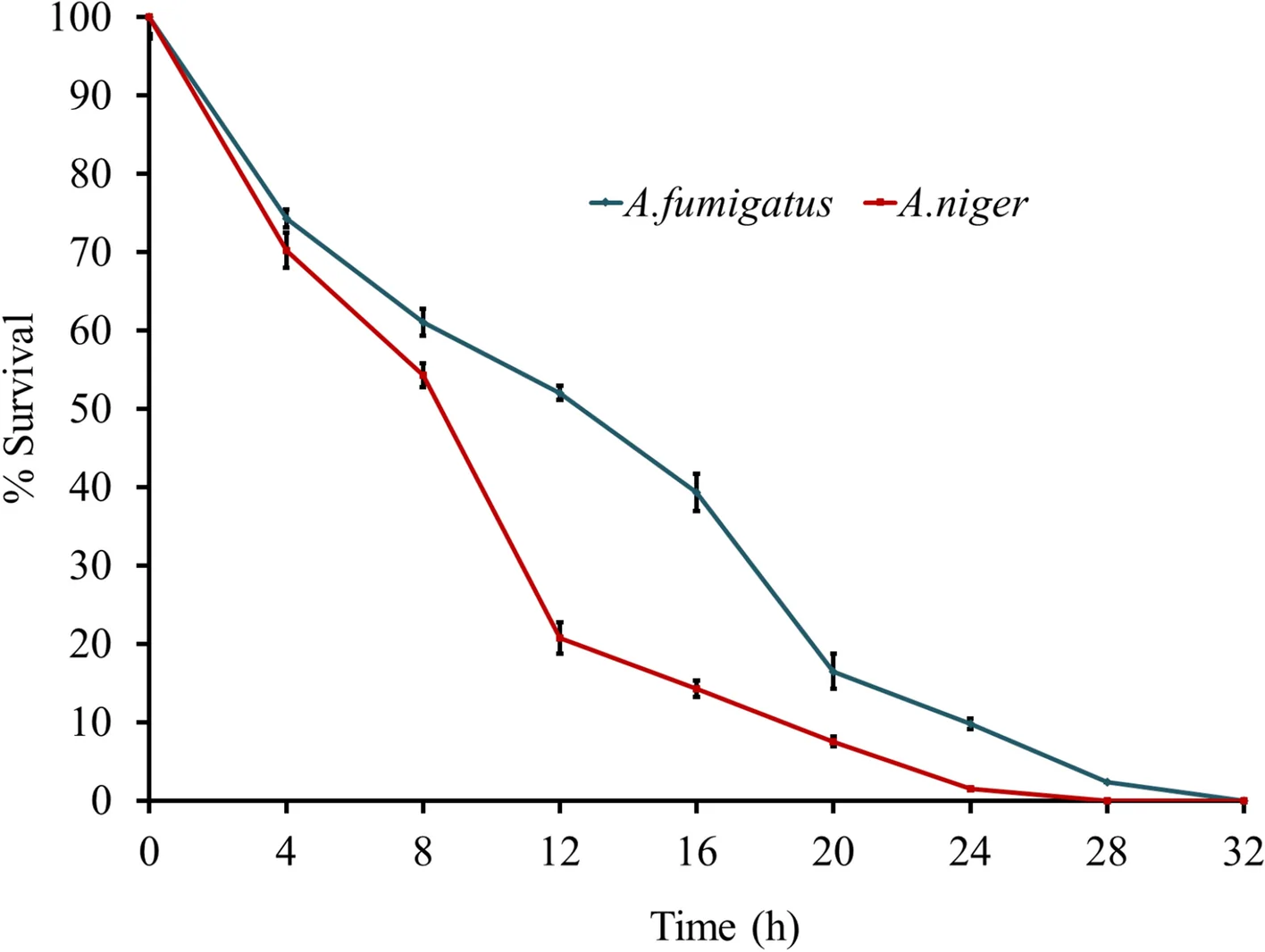
Killing kinetics of fungus by temporin-SHf. Fungal strains: *A.fumigatus* or *A.niger* were treated with the MFC concentration of temporin-SHf

**Fig. 5:**
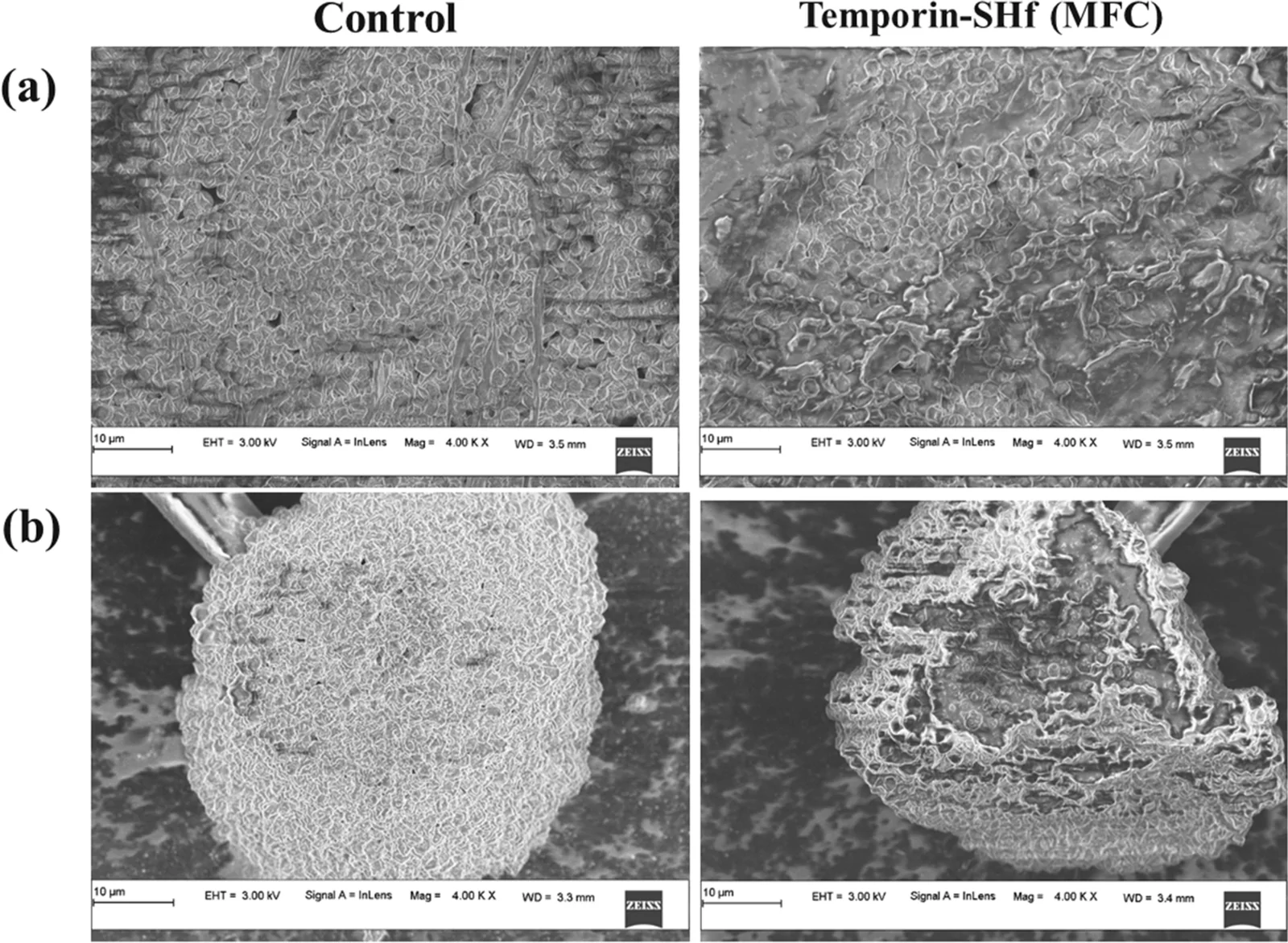
SEM images of fungal strains: **a** *A.fumigatus* or **b** *A.niger* in control and treated with the MFC concentration of temporin-SHf for 24 h

Fungi have a chitin cell wall and an ergosterol inner membrane. The cell wall components are unique and constitute excellent drug targets (Hasim and Coleman 2019). The antifungal activity could be essential to amphibians, considering that the *B. dendrobatidis* was identified as the culprit for amphibian decline (Berger et al. 1998). The non-declining amphibians have more effective AMPs than those declined species in the same niche. Amphibian peptides such as magainin 2, peptide glycine-leucine-amide (PGLa), temporin-1P, brevinins, dermaseptin-L1, phylloseprin, and ranatuerins inhibit *B. dendrobatidis *(Rollins-Smith 2009). Antifungal assay reveals that the temporin-SHf was slightly fungicidal; the MFC values indicated that the peptide is weakly active against *A. niger* and *A. fumigatus* (Table 3). Temporin-SHf can act against *A. flavus* (> 200 µM) (Abbassi et al. 2010). Its activity against fungal strains for about 27–32 h (Fig. 4) and SEM images of fungal strains of *A.fumigatus* or *A.niger* in control and treated groups with the MFC concentration of temporin-SHf for 24 h (Fig. 5) indicate that amphibian peptides are likely to kill fungi by damaging membranes (Rinaldi et al. 2002; Bondaryk et al. 2017). Temporin-SHf was initially discovered as an antimicrobial peptide with broad-spectrum microbiocidal (Abbassi et al. 2010). As a positive control, it is interesting that this study's antimicrobial activity data (Tables 2, 3) for temporin-SHf align with the previous findings (Abbassi et al. 2010). In addition, the MIC of this peptide against Gram-positive and Gram-negative bacteria was consistent with earlier reports (Table 2), indicating that the temporin-SHf is bactericidal. However, the peptide was weakly active or without activity against the fungus (Table 3). This peptide's short length, compositional simplicity, and broad-spectrum antimicrobial activity make it an attractive candidate for developing new antimicrobial agents, and it can be produced efficiently by chemical synthesis for clinical use as antimicrobials.

**Table 3:** Antifungal activity of temporin-SHf

Fungal strains	MFC (μM)
*Aspergillus niger*	429.10 ± 2.216
*A. fumigatus*	488.89 ± 3.317

MFC concentrations represent the minimum dose required to kill the entire fungus

### Hemolysis activity of temporin-SHf

**Fig. 6:**
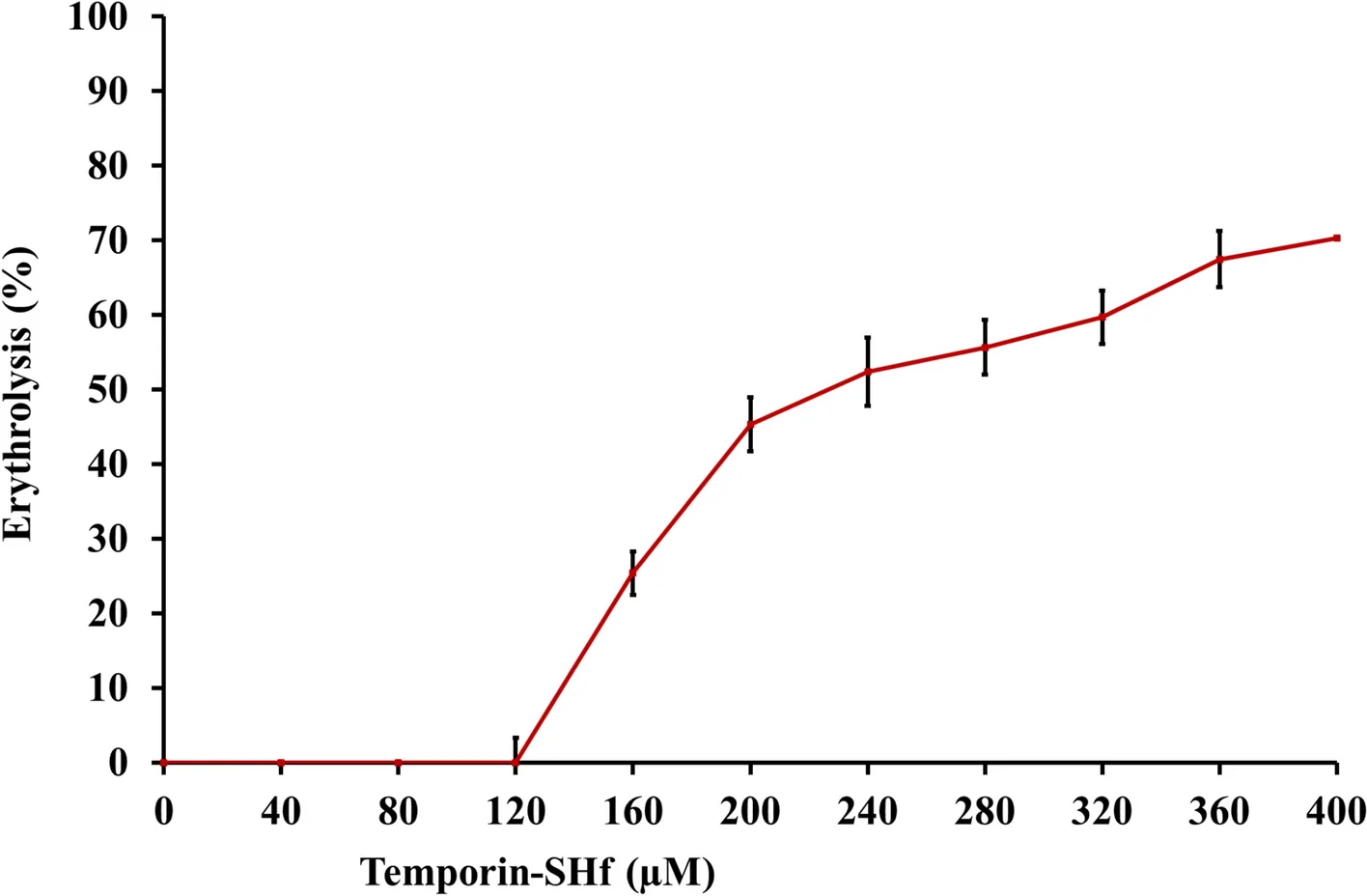
Hemolytic activity of temporin-SHf

Temporin-SHf was non-hemolytic against human erythrocytes up to 120 μM. The (HL_50_) 50% erythrolysis was only at 267.97 μM temporin-SHf concentration (Fig. 6), far above the MIC/MFC/IC_50_ values determined for the bacterial/fungal strains/human cancer cells. Previous studies also mentioned that temporin-SHf showed no hemolytic action against human erythrocytes (Abbassi et al. 2010). Usually, amphibian peptides do not have substantial hemolytic activity against human erythrocytes. For example, the HC_50_ of ranatuerin-2 (35 μM), ranatuerin-2 Ga (35 μM), ranatuerin-2BYb (> 200 μM), and ranatuerin-2VLb (> 200 μM) (Goraya et al. 1998; Conlon et al. 2009). Temporins have α-helix confirmation and showed low/no hemolytic activity against rabbit erythrocytes and human erythrocytes (Diao et al. 2012; Wang et al. 2012). The mechanism of amphibian peptides' hemolytic action is not well understood. But it might depend on conformation, positive charge, hydrophobicity, and amphipathicity (Gagnon et al. 2017). The insignificant hemolytic activity of the temporin-SHf (Fig. 6) might be due to its secondary structure features (Table 1).

### Temposrin-SHf caused cytotoxicity in human cancer cells

**Fig. 7:**
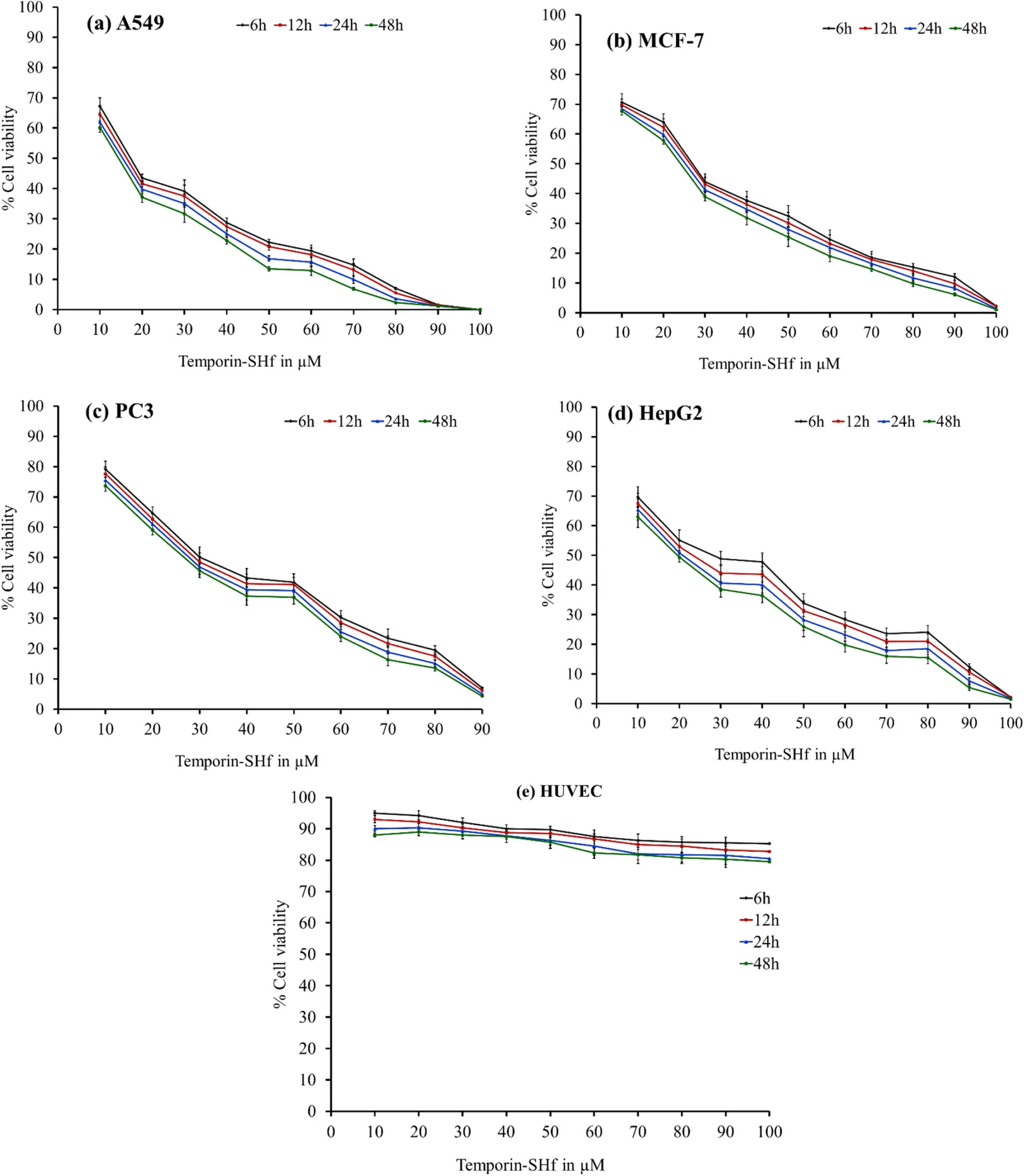
Temporin-SHf caused cytotoxicity in human cancer cells, and HUVEC was determined by MTT assay

Anticancer drugs explicitly targeting cancer cells and killing them with high efficiency are urgently needed. The cationic AMPs with additional tumoricidal properties, called cationic anticancer peptides (ACPs), have emerged as promising agents offering several advantages over conventional anticancer drugs (Oelkrug et al. 2015). The cytotoxic and cell-selective potential of temporin-SHf was analyzed using four human cancer cell lines and normal HUVEC (Table 4; Fig. 7). The reduced cell viability observed in temporin-SHf treated with A549, MCF-7, HepG2, and PC3 cancer cells (Fig. 7) exhibits their tumoricidal property. The IC_50_ values of temporin-SHf treated against various cancer cells (Table 4) showed that temporin-SHf effectively kills cancer cells like other amphibian AMPs. A549 cancer cells are more sensitive to temporin-SHf than the other cancer cells, indicating that it effectively kills lung cancer cells. This difference in sensitivity may be attributed to differences in cell membrane composition, fluidity, and surface area between these human cancer cell lines (Hoskin and Ramamoorthy 2008).

**Table 4:** Cytotoxicity (IC_50_) of temporin-SHf on human cancer cells

Human cancer cells	IC_50_ (in µM)			
	6 h	12 h	24 h	48 h
A549	27.71 ± 1.150	26.27 ± 1.054	24.03 ± 0.945	21.85 ± 0.927
MCF-7	35.77 ± 1.854	34.45 ± 1.645	32.76 ± 1.528	30.79 ± 1.197
PC-3	40.10 ± 0.721	38.62 ± 0.658	36.67 ± 0.729	35.05 ± 0.673
HepG2	37.95 ± 1.111	35.31 ± 1.248	32.64 ± 1.350	30.32 ± 1.364

Temporin-SHf exhibits cytotoxicity to human cancer cells but not to non-tumorigenic cells (primary cell HUVEC), indicating that temporin-SHf is nontoxic to cells unassociated with cancer and cancer-cell selective (Fig. 7). Peptide's selective killing of cancer cells is unclear. However, it may be due to the cell membrane composition and the distribution of phospholipids, which could determine the cell selectivity and susceptibility to lysis (Hoskin and Ramamoorthy 2008). In this study, temporin-SHf showed antimicrobial and anticancer activity, possibly due to its hydrophobicity, very short amino acid residue, and positive charge (Table 1). Many forces are involved in the interaction of peptides with cell membranes, such as electrostatic attraction forces, hydrophobic interactions, and van der Waals interactions (Papo and Shai 2003). The peptide's strategy to kill cancer cells more selectively than normal mammalian cells might be the electrostatic attraction between the number of negatively charged phospholipid molecules on cancer cells, and the positively charged peptides are believed to play a significant role in the selective binding and disruption of cancer cell membranes (Riedl et al. 2011). The negatively charged phospholipids: anionic phosphatidylserine and phosphatidylethanolamine, or a higher number of microvilli, increase the surface area of their membranes, which is unusual in normal cells (Papo and Shai 2003; Tornesello et al. 2020). The surfaces of normal mammalian cell membranes are mainly composed of neutral zwitterionic phospholipids and sterols (Chen et al. 2003). Such a bacteria-like membrane may provide a molecular basis for the cell selectivity of cationic peptides (Dos Santos et al. 2017). Studies have demonstrated that cationic peptide aurein 1.2 interacts with anionic lipid membranes and enters the T98G glioblastoma cells (Dennison et al. 2017). It binds explicitly to anionic *O*-, *N*-glycoproteins, and gangliosides molecules on MX-1 and MCF-7 breast cancer cells (Han et al. 2013). The presence of anionic molecules such as glycoproteins and glycolipids on the surface of cancer cells and the negatively charged phospholipids on the outer cell membrane has been proposed as the selection mechanisms for the targeted action of ACPs toward cancer cells (Armbrecht et al. 2017). Therefore, glycosylation in cancer cells plays a vital role in the anticancer activity of peptides, which may partly explain their cancer cell-selective toxicity. Available cancer drugs slander their efficiencies in front of the MDR of cancer cells (Al-Mugotir et al. 2021; Tiek and Cheng 2022). Temporin-SHF showed selectivity towards microbes, and cancer cells, not normal mammalian cells (Fig. 7). This naturally occurring cationic peptide might be a promising new anticancer drug candidate that can overcome the MDR by cancer cells and the adverse side effects.

### Effect of temporin-SHf on human cancer cell viability

**Fig. 8:**
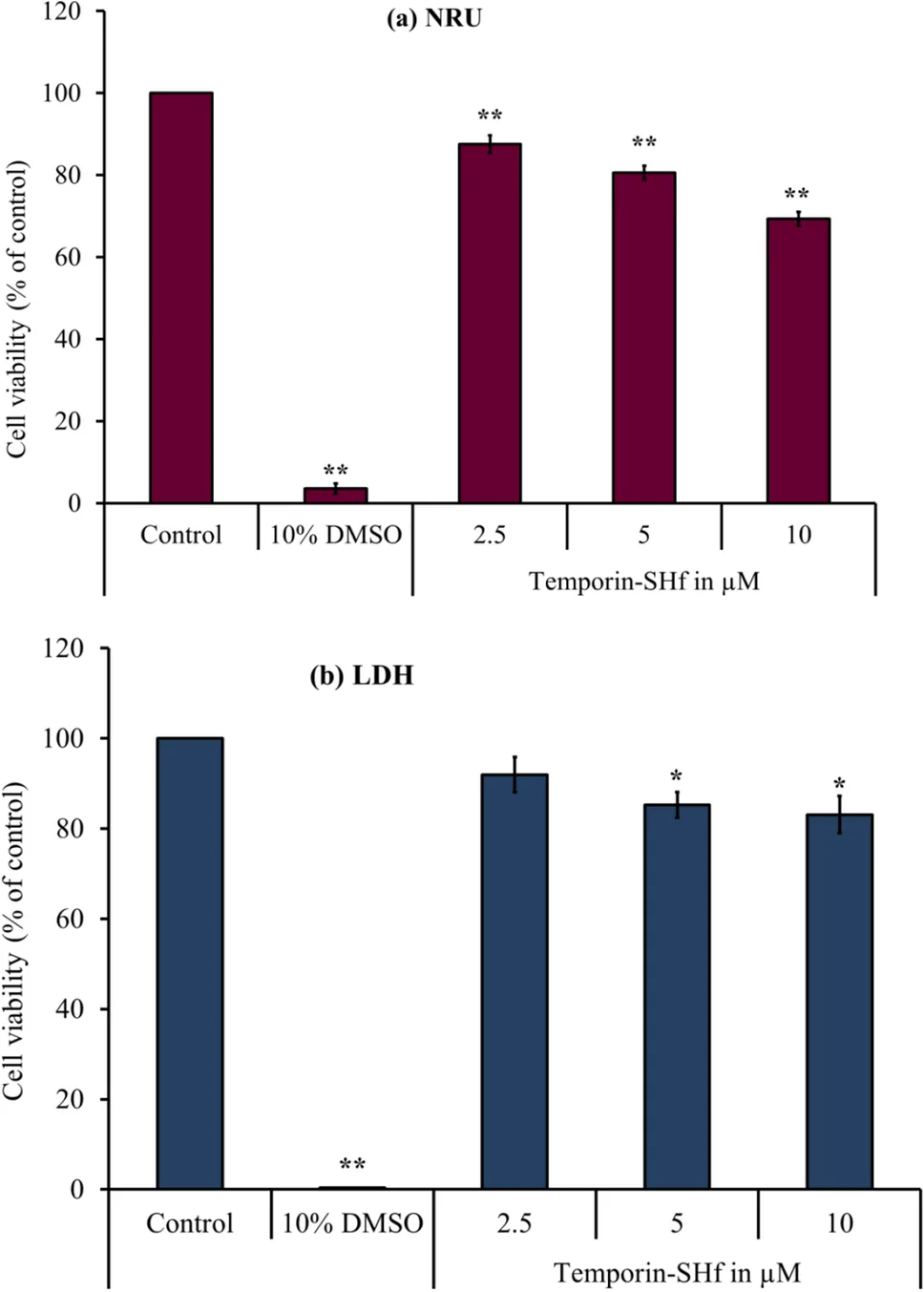
NRU and LDH assay were conducted in A549 cancer cells treated with temporin-SHf and control groups. **a** NRU assay determined cell viability as a loss of lysosomal integrity, and **b** LDH assay determined cell viability as cell membrane damage. Values are significant compared to control at *p < 0.05; **p < 0.01. The histogram bar does not have any symbols that are insignificant at *p* > 0.05

The NRU assay is an accurate and reliable technique used to evaluate the toxicity of compounds (Repetto et al. 2008). It determined cell viability to assess temporin-SHf effects on A549 cells' lysosomal integrity. Cells maintained an apparent viability rate during exposure with a lower concentration of temporin-SHf for 24 h. The higher concentrations of temporin-SHf marked a significant decline in cell viability (*p* < 0.01), which showed its cytotoxic potential (Fig. 8a). In control, there was an insignificant increase in the rate of release of cytosolic enzyme LDH, indicating the preserved plasma membrane integrity. The LDH activity significantly differed between the control and those treated with temporin-SHf for 24 h (Fig. 8b), indicating that temporin-SHf causes cell membrane damage and releases LDH. However, A549 cancer cells treated with temporin-SHf for 24 h significantly increased the percentage of cytotoxicity, suggesting that temporin-SHf induces cancer cell death. Like this, LfcinB, a cationic AMP isolated from cow's milk triggers mouse fibrosarcoma and human neuroblastoma cells to die primarily via necrosis (Eliassen et al. 2006). Temporin L was cytotoxic to three human cancer cell lines (Hut-78, K-562, and U-937), causing necrosis-like cell death (Rinaldi et al. 2002). Brevinin-1RL1, a cationic α-helical AMP isolated from frog *R.limnocharis* skin secretions, inhibits cell viability through necrosis (Ju et al. 2021). It also demonstrated that Brevinin-1RL1 interacts with the lipids or particular proteins on the plasma membrane, inducing apoptosis and necrosis to suppress tumor cells and exert anticancer activity. It is clear that temporin-SHf causes necrosis or apoptosis in cancer cells due to its cationic nature; it might bind to lipid membranes to form pores that permeabilize the cell membrane (Abbassi et al. 2010).

### Temporin-SHf inhibits cancer cell proliferation

**Fig. 9:**
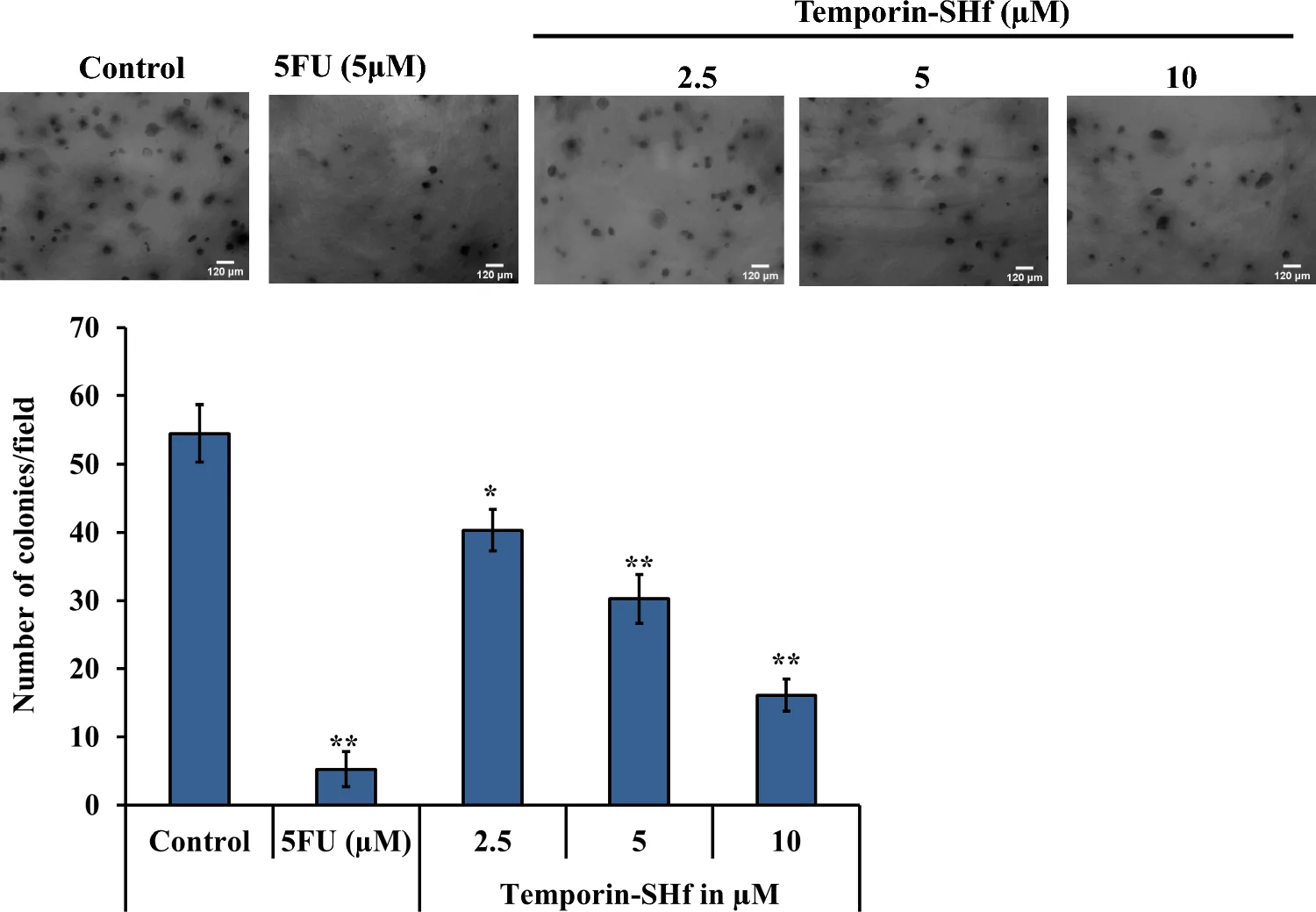
Effect of temporin-SHf on A549 cells colony formation. Representative plates of A549 cell colonies in respective groups. The histogram shows the number of A549 cell colonies in respective groups. Values are significant compared to control at *p < 0.05; **p < 0.01

A soft agar assay was employed to evaluate the anchorage-independent growth of cancer cells, a hallmark of malignant transformation (Borowicz et al. 2014). A significantly reduced number of colonies was noticed in A549 cells cultured in soft agar with 5-FU (positive control) (Fig. 9; *p* < 0.01), showing its cancer cells' antiproliferation. 5-FU can block thymidine formation by inhibiting the thymidylate synthase required for DNA synthesis (Peters et al. 2002). AMPs isolated from amphibians are also exhibiting inhibition of cancer cell proliferation. Dermaseptin B2 isolated from *Phyllomedusa bicolor* inhibits PC3 and MDA-MB231 cancer cell colony formation in soft agar (Wang et al. 2012). PsT-1, a tryptophyllin peptide from the skin secretion of the waxy monkey leaf frog, *Phyllomedusa sauvagei* inhibits proliferation in prostate cancer cells (Wang et al. 2013). Bombinin-like peptide (BLP-7) and bombinin H-type peptide (Bombinin H-BO) from the skin secretion of *B. orientalis* inhibits HepG2/SK-HEP-1/Huh7 human hepatoma cell proliferation (Zhou et al. 2018). Dermaseptin-PS4 (Der-PS4) isolated from the skin secretion of the waxy monkey tree frog, *P. sauvagii* showed antiproliferative capacity against cancer cell lines (Chen et al. 2018). Brevinin-1GHd inhibited the proliferation of human cancer cell lines (Jiang et al. 2020). A similar significant cancer cell growth inhibition was observed when A549 cells were cultured in soft agar treated with temporin-SHf (Fig. 9; *p* < 0.01), while the mechanism remained unclear.

### Temporin-SHf inhibits the migration of cancer cells

**Fig. 10:**
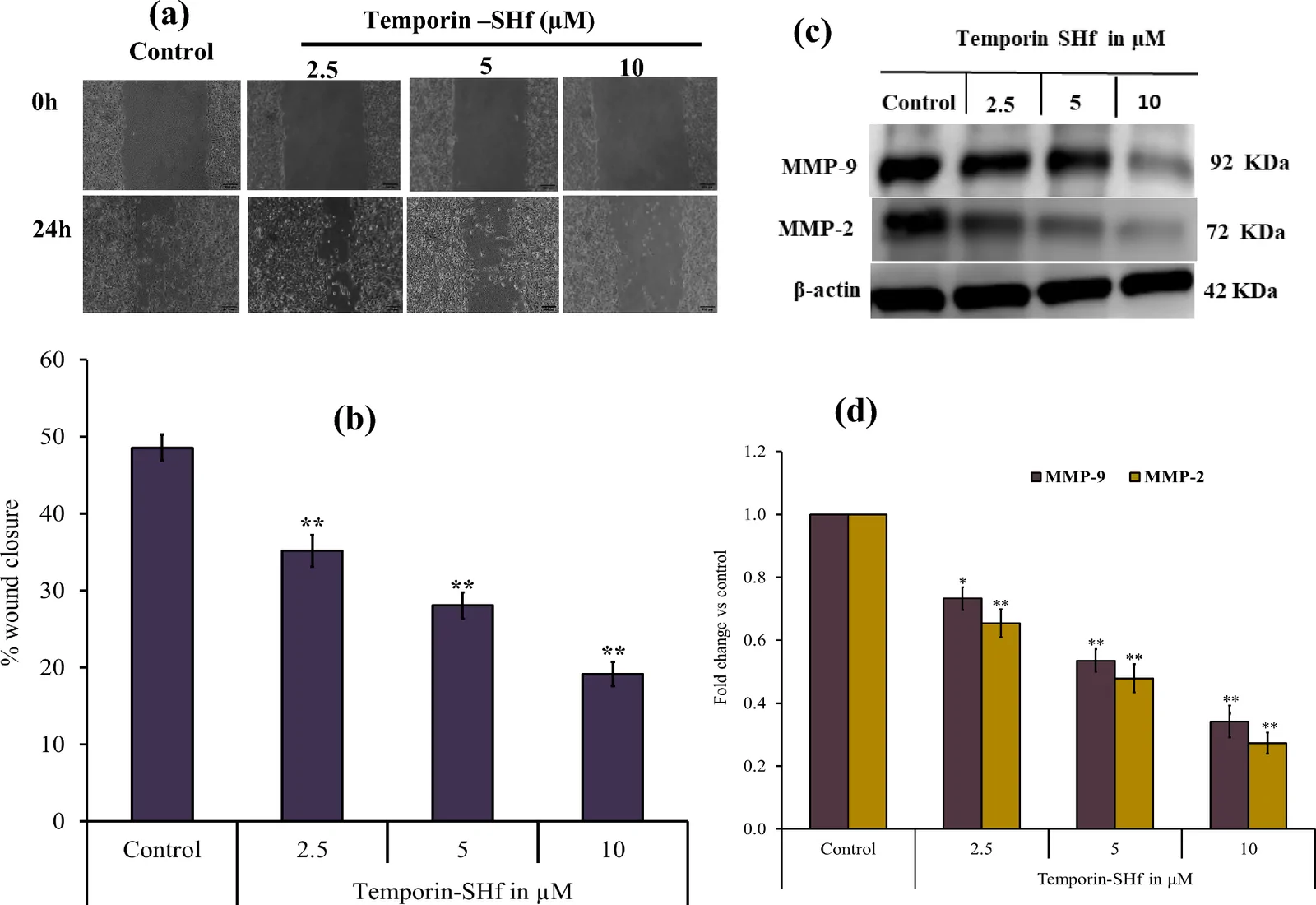
Temporin-SHf inhibits A549 cancer cell migration. **a** Tumor cell scratch assay. **b** The % of wound closure in control and temporin-SHf treated groups. **c** MMP-9 and MMP-2 protein expression analyzed by Western blot and d The relative level of MMP-9 and MMP-2 protein expression in respective groups. Values are significant compared to control at *p < 0.05; **p < 0.01

Cancer progression involves cell migration, invasion, and adhesion (Pijuan et al. 2019). Therefore, a simple, reproducible tumor cell scratch assay was employed to determine the effectiveness of temporin-SHf against cancer cell migration. The A549 cell monolayer treated with temporin-SHf showed a highly significant reduction of scratch healing (wound closure) (*p* < 0.001; Fig. 10a, b), indicating that the temporin-SHf treatment prevents cancer cell migration. A similar type of prevention of cancer cell migration was demonstrated by cinobufacini and arenobufagin, isolated from toad *B. arenarum* (Wang et al. 2020; Zhao et al. 2020). The matrix metalloproteinases MMP-2 and MMP-9 are also known as A and B gelatinases, which regulate epithelial cell migration (Chen and Parks 2009). Both are involved in early carcinogenesis events, tumor growth, invasion, and metastasis. The significantly reduced MMP-2 and MMP-9 protein expression in A549 cells were treated with temporin-SHf (*p* < 0.01; Fig. 10c, d), indicating that it prevents cancer cell migration by inhibiting the matrix metalloproteinases expression.

### Temporin-SHf anti-angiogenesis nature

**Fig. 11:**
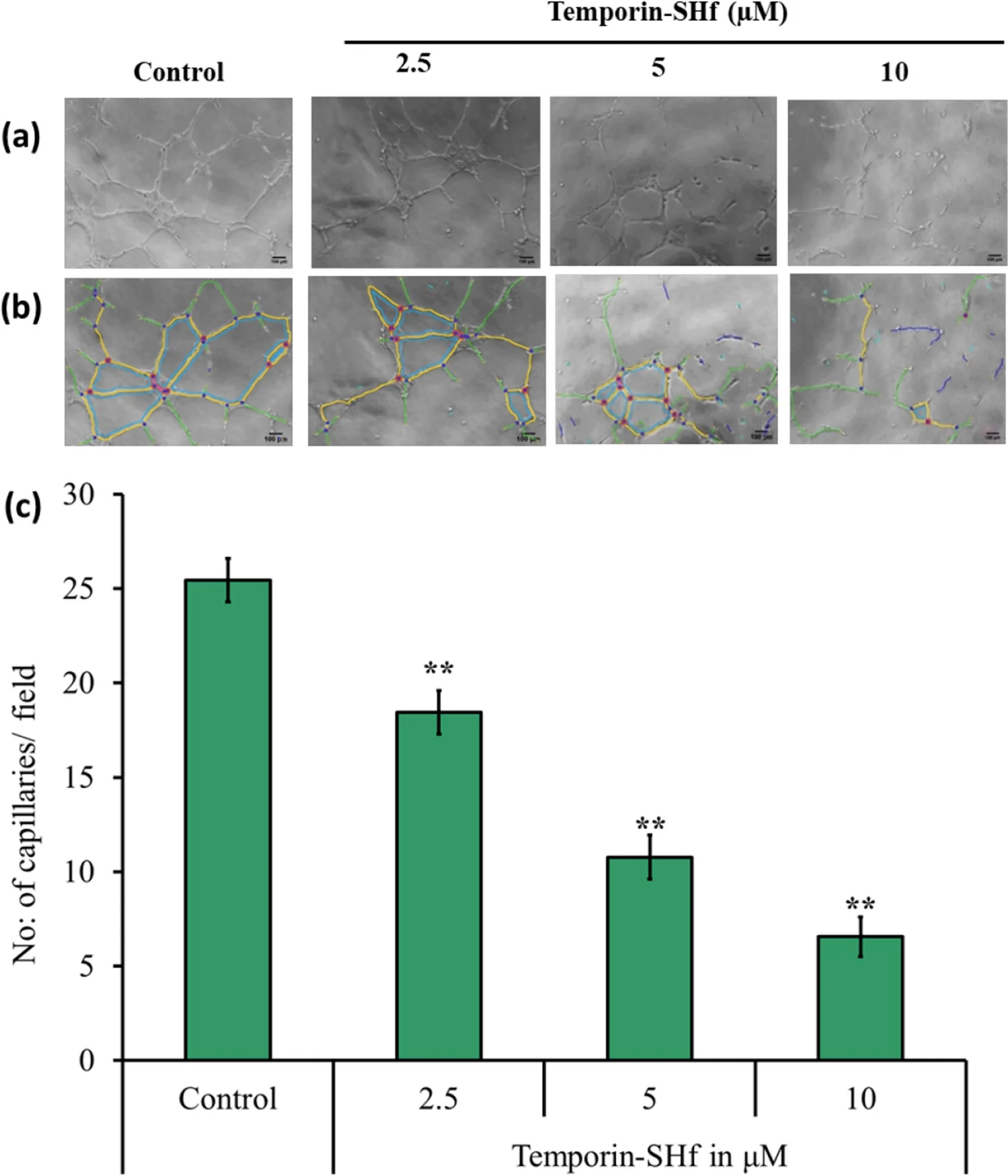
Angiostatic effect of temporin-SHf. **a** HUVEC treated with PBS (control) and different concentrations of temporin-SHf **b** angiogenesis analyzed by ImageJ software (green = branches; cyan = twigs; magenta = segments; orange = master segments; blue sky = meshes; red surrounded by blue = nodes surrounded by junctions symbol. **c** No. of capillaries/fields in experimental groups. Values are significant compared to control at **p < 0.01

**Fig. 12:**
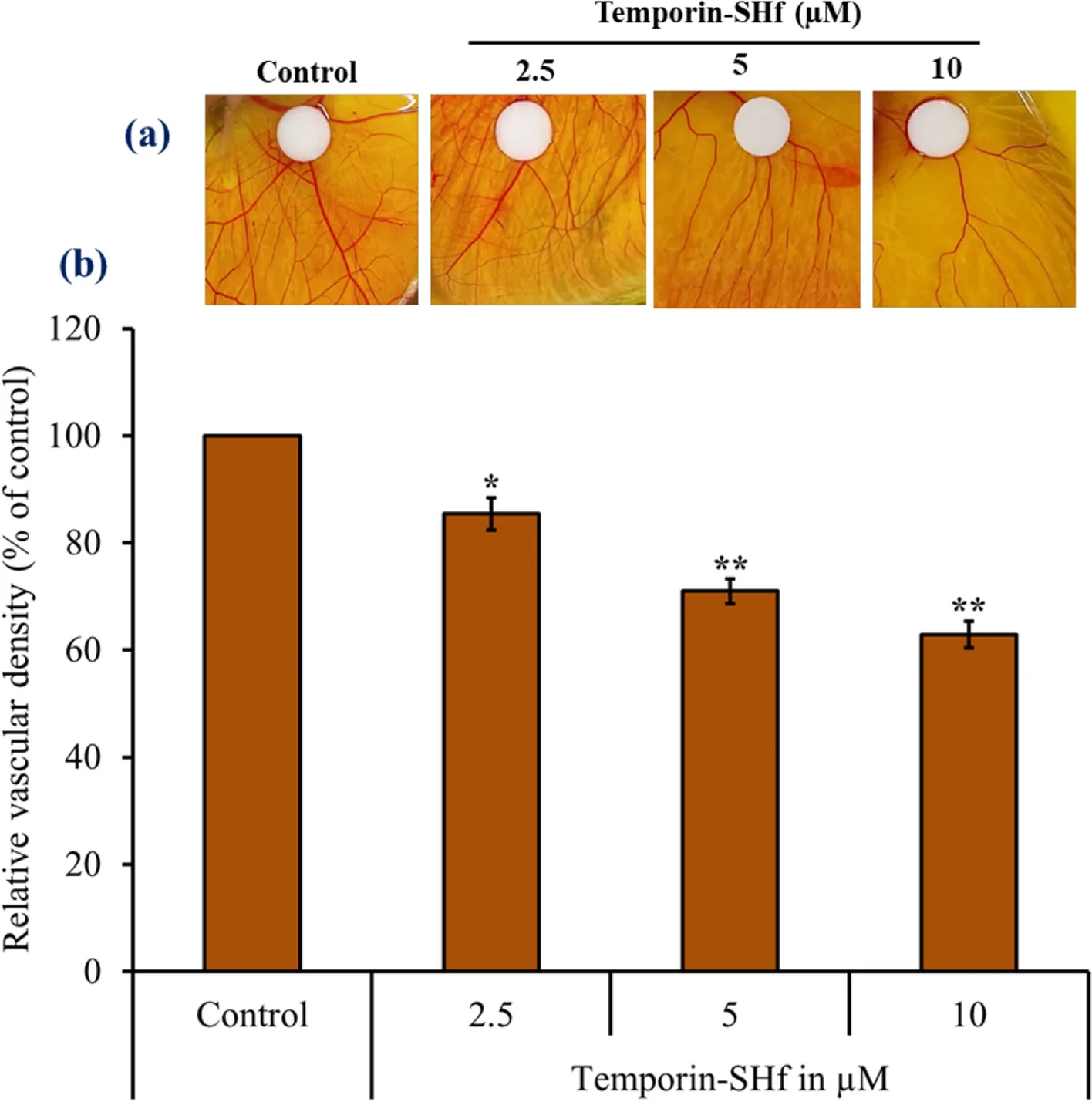
Anti-angiogenic effects of temporin-SHf. **a** CAMs exposed to disc soaked in PBS (control) and different concentrations of temporin-SHf. **b** Relative vascular density in treatment groups. Values are significant compared to control at *p < 0.05; **p < 0.01

**Fig. 13:**
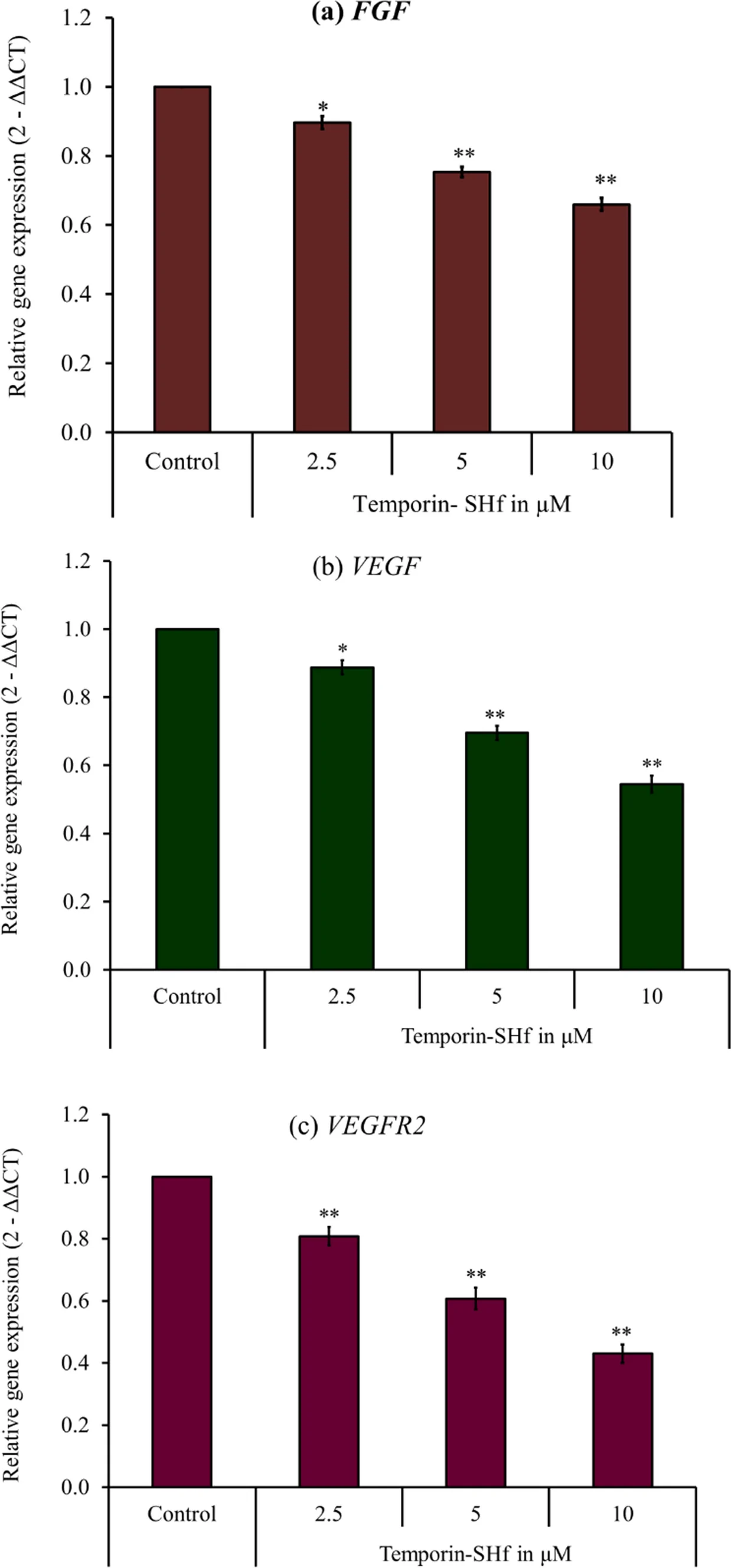
Temporin-SHf induced anti-angiogenic gene expression in the CAMs. Values are significant compared to control at *p < 0.05; **p < 0.01

The angiostatic nature of temporin-SHf was analyzed using an in vitro angiogenesis assay. In the control group, HUVEC underwent differentiation and formed capillary networks on matrigel, which confirms in vitro angiogenesis. Significantly reduced HUVEC differentiation, diminished tubular network formation, and the number of capillaries in temporin-SHf-treated groups (*p* < 0.001; Fig. 11) evidenced that this peptide inhibits the growth of blood capillaries. Other studies also demonstrated that amphibian peptides dermaseptin-B2 inhibits ABAE endothelial cells differentiation, arenobufagin inhibits HUVEC tube formation (Wang et al. 2020; van Zoggel et al. 2012; Xia et al. 2019). Significantly reduced blood vessel density recorded in various concentrations of temporin-SHf-treated groups than the control in vivo chicken embryo chorioallantoic membrane (CAM) (*p* < 0.01; Fig. 12) showed the anti-angiogenic nature of the peptide. Supporting this, other amphibian peptides arenobufagin and anginex (βpep-25), also suppressed the growth of micro capillaries in CAM (Griffioen et al. 2001). Tumor cells can induce vascular endothelial cells to form new blood vessels through increased vascular endothelial growth factor (VEGF) expression, promoting tumor growth and metastasis. The relative angiogenic gene expression of fibroblast growth factor (*FGF*), *VEGF*, and VEGF receptor 2 (*VEGFR2*) was significantly down-regulated in CAMs treated with temporin-SHf (Fig. 13; *p* < 0.01), indicating their potential anti-angiogenesis nature. As like, an amphibian Chan Su peptide inhibited the protein expression of VEGF165 and Ras, exerting anti-angiogenic effects by suppressing the VEGF165-VEGFR2-Ras signaling pathway (Xia et al. 2019). The αAL14, a synthetic peptide, inhibits tube formation in HUVEC by suppressing the activity of VEGFR2 (Kim et al. 2016). Temporin-1CEa was found to prevent the formation of VEGF in human melanoma A375 cells, thus inhibiting the formation of new blood vessels at an IC_50_ value of 18.2 μM (Wang et al. 2016). The possible explanation for the angiostatic activity of the temporin-SHf is unknown. Temporin-SHf does kill tumor cells and inhibits neovascularization; hence, it has minimal side effects on normal cells. Therefore, AMPs of this kind have good prospects for clinical application.

### Apoptotic activity of temporin-SHf

**Fig. 14:**
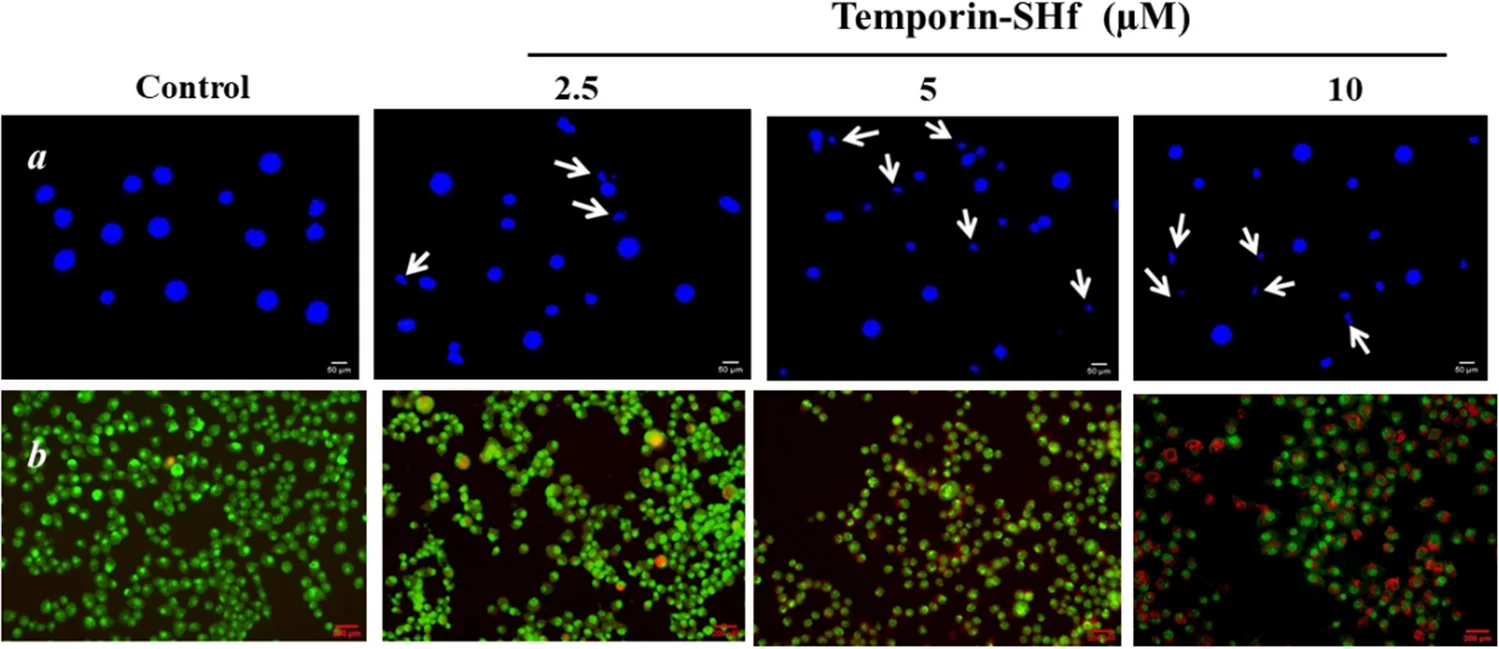
Temporin-SHf induces apoptosis in A549 cells. **a** A549 cells stained with Hoechst 33342 nuclear stain. **b** AO/EB in control and different concentrations of temporin-SHf treated groups

**Fig. 15:**
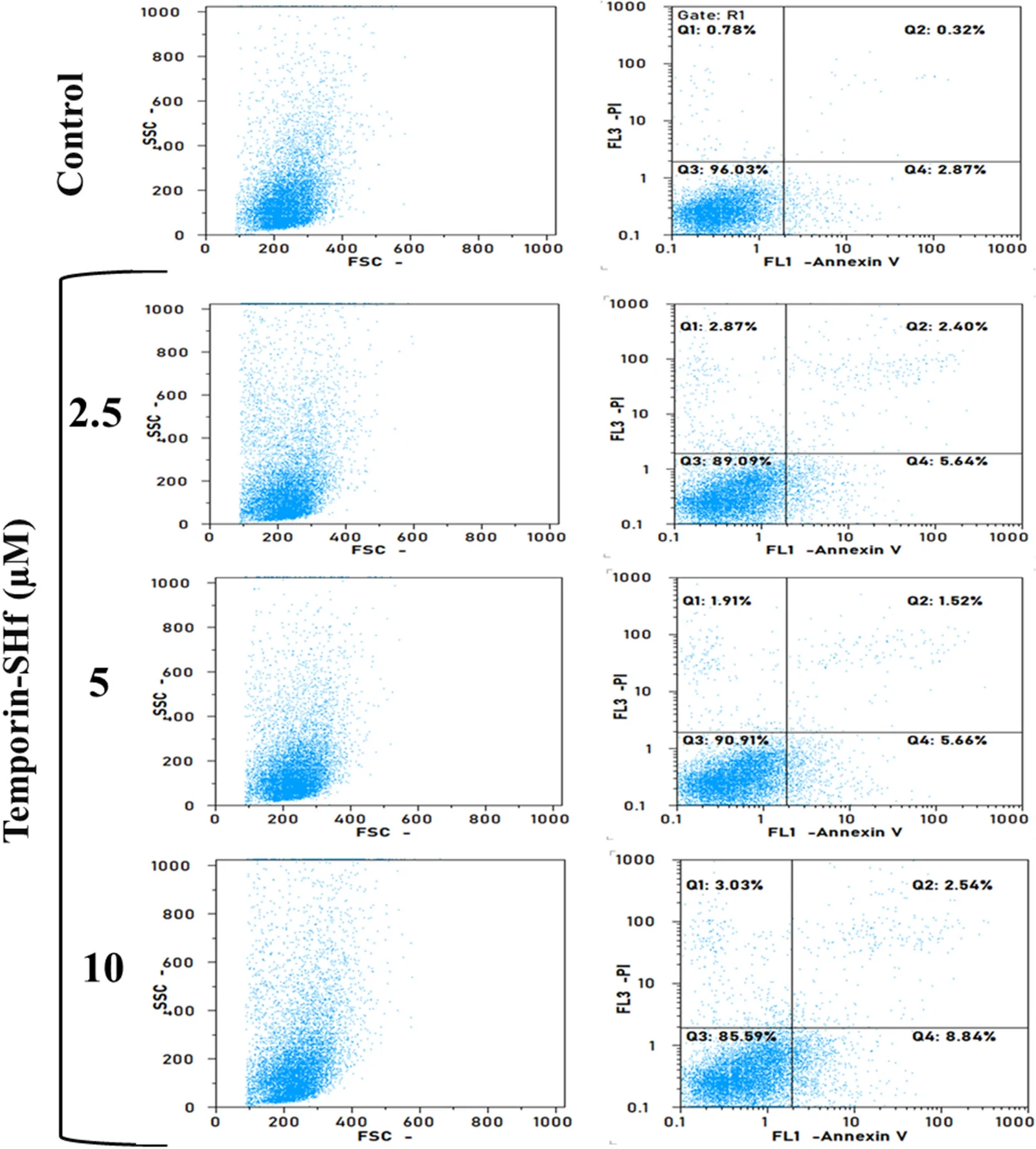
Temporin-SHf induced apoptosis in cancer cells analyzed by flow cytometry after Annexin V/PI staining in control and temporin-SHf treated cells

Temporin-SHf preferential inhibition of cancer cell proliferation mechanism is unclear; hence, we studied the temporin-SHf-induced cancer cell death process. The temporin-SHf treatment not only reduced the density of A549 cells in a dose-dependent manner (Fig. 14) but also changed the cancer cells' shape. The changes in chromatin morphology, such as condensation and fragmentation, were noticed in the Hoechst 33342 nuclear stained A549 cells, which were treated with temporin-SHf for 24 h. Healthy cells' nuclei were spherical in the controls (Fig. 14a). The non-apoptotic green cells in the control groups and red apoptotic cells in the temporin-SHf-treated groups in dual AO/EB fluorescent stained A549 cells (Fig. 14b). Therefore, it is clear that temporin-SHf-induced cancer cell death might be through apoptosis mechanism. In order to confirm temporin-SHf induces apoptosis in cancer cells, performed with Annexin V and Propidium Iodide stained flow cytometry analysis. The obtained data, showing an increased percentage of early apoptotic cells in temporin-SHf vs. control cells (Fig. 15).

**Fig. 16:**
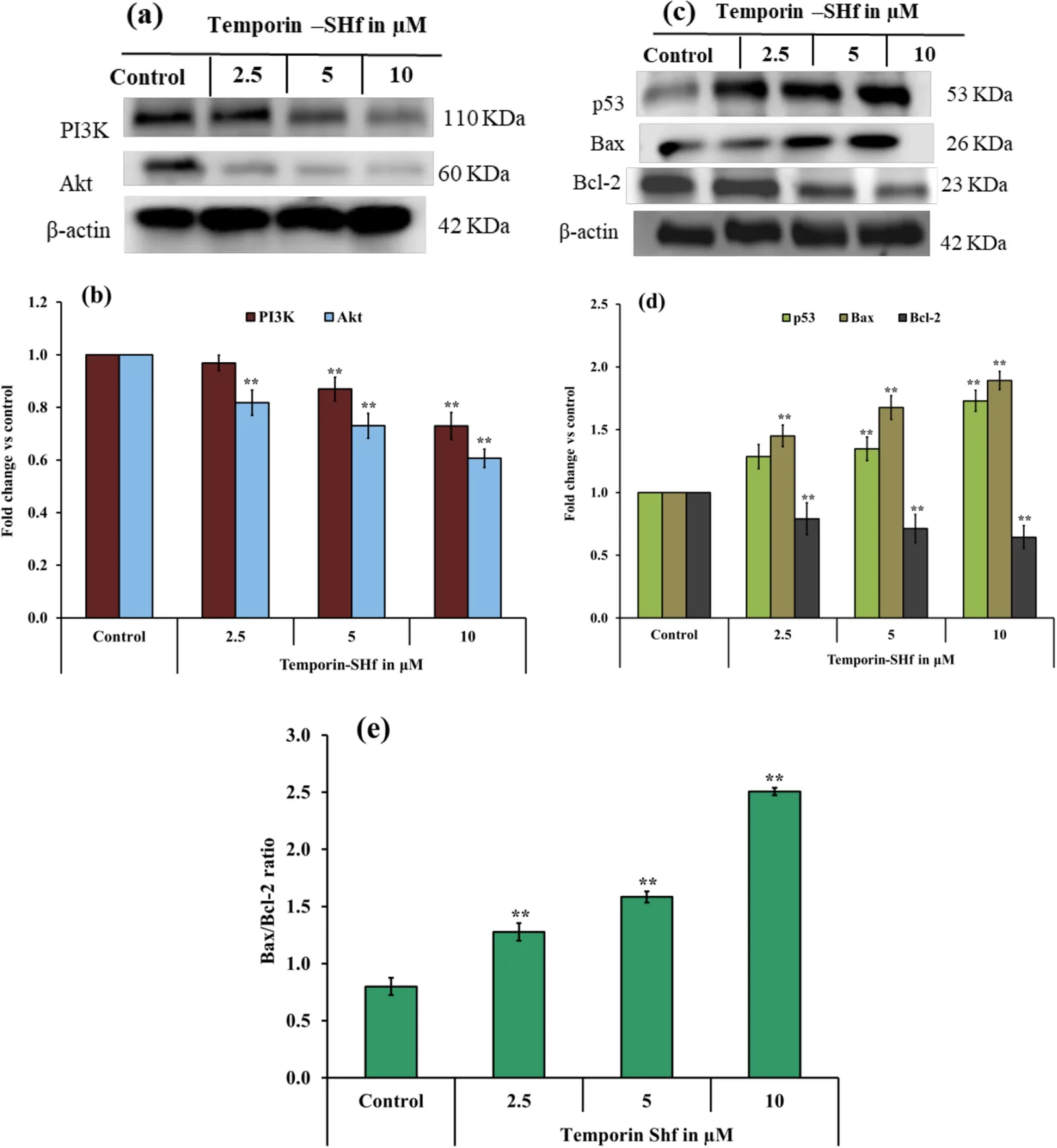
Temporin-SHf induced apoptosis in A549 cancer cells. **a** and **c** western blot analysis of apoptosis proteins. **b** and **d** The relative expression of apoptosis proteins. **e** Bax/Bcl-2 ratio in control and temporin-SHf treated groups. Values are significant compared to control at **p < 0.01. The histogram bar does not have any symbols that are insignificant at *p* > 0.05

The current study found that temporin-SHf can induce apoptosis in A549 cells by down-regulating the master proteins Akt and PI3K (Fig. 16). Akt is crucial for cell metabolism, growth, proliferation, and survival. Its activation is controlled by a multi-step process that involves PI3K (Hemmings and Restuccia 2012). Akt can inactivate pro-apoptotic factors such as Bad and procaspase-9, thus inhibiting apoptosis (Porta et al. 2014). Up-regulation of p53 can up-regulate Bax and down-regulate Bcl-2, resulting in a significant increase in the Bax/Bcl-2 ratio, a driving force for apoptosis (Ramadan et al. 2019). The balance of pro- and anti-apoptotic proteins of the Bcl-2 family is known to regulate cell survival and apoptosis. Bcl-2, an anti-apoptotic protein, plays a vital role in the resistance of cancer cells to chemotherapy or radiation therapy (García-Aranda et al. 2018). The significantly increased Bax/Bcl-2 ratio in the temporin-SHf-treated A549 cancer cells than the control group (Fig. 16e) confirms that the apoptosis pathway is involved in cancer cell death.

**Fig. 17:**
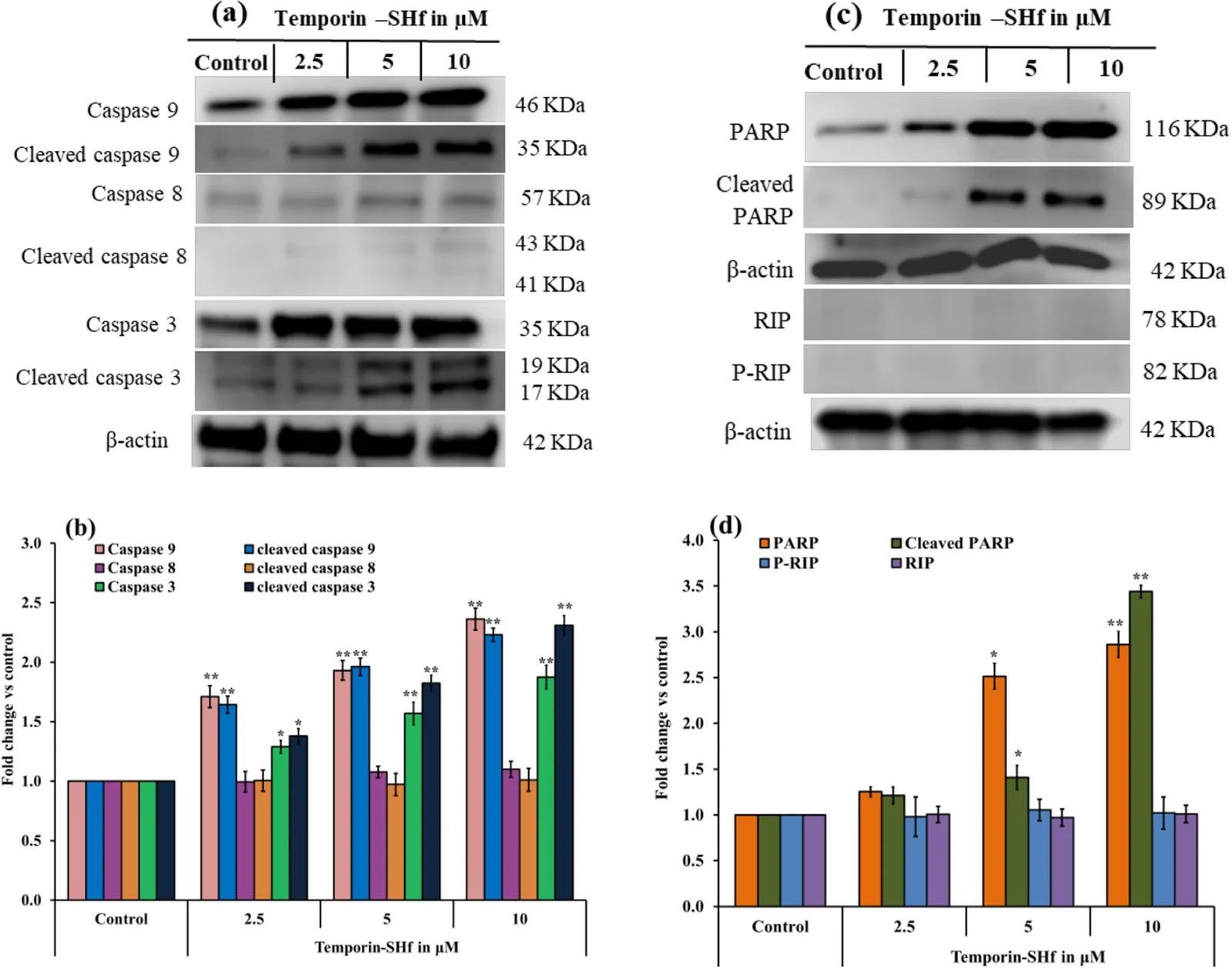
Temporin-SHf induced apoptosis in A549 cancer cells. **a** and **c** western blot analysis of apoptosis proteins. **b** and **d** The relative expression of apoptosis/necroptosis proteins. Values are significant compared to control at *p < 0.05; **p < 0.01. The histogram bar does not have any symbols that are insignificant at *p* > 0.05

**Fig. 18:**
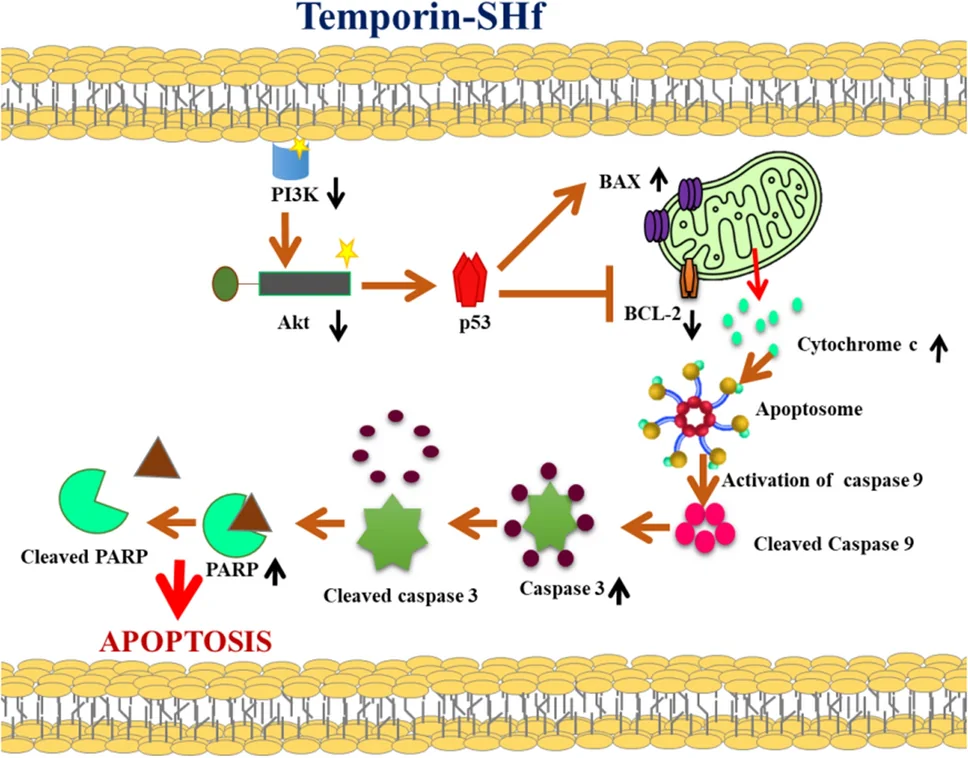
The mechanism of action of temporin-SHf

Initiation of apoptosis required caspase cascade events. Caspases are generally crucial mediators of apoptosis and regulate intrinsic mitochondrial pathways and extrinsic death receptor transduction pathways according to the caspases involved. The extrinsic induces the cleavage of caspase 8, while the intrinsic pathway activates caspase 9, leading to the subsequent activation of caspase 3 and poly (ADP-ribose) polymerase (PARP) to induce apoptosis (Lossi 2022). PARP is a DNA repair nuclear protein that can cleave by activated caspases 3 and 7 in caspase-dependent apoptosis. Once PARP is cleaved, it loses its function, suppressing DNA repair and thus leading to apoptosis (Mashimo et al. 2021). The cleavage of caspases was detected to verify whether both pathways are involved in temporin-SHf-induced apoptosis in cancer cells. The extrinsic apoptosis indicator caspase-8 was not cleaved, and the intrinsic caspase-9 was cleaved to activate caspase-3 and PARP (Fig. 16c), confirming that the intrinsic mitochondrial pathway is involved in temporin-SHf-induced apoptosis. This study demonstrates that temporin-SHf-induced inhibitory cancer cell proliferation is a caspases-mediated intrinsic apoptosis pathway. The non-expression of necrosis markers such as P-RIP and RIP (Fig. 17c) also confirms the non-involvement of necrosis pathways. In addition, the insignificant release of LDH in lower concentrations of temporin-SHf (Fig. 8) indicated necrosis pathway was not involved in the cytotoxicity of the peptide. These results demonstrate temporin-SHf-induced caspase-dependent apoptosis through intrinsic mitochondrial pathways. Further studies are required to understand the mechanism of induction of apoptosis by temporin-SHf; inducing cancer cell apoptosis is essential for ACPs to exert an antitumor effect. The schematic representation of the apoptotic activity of the temporin-SHf is provided in Fig. 18.

## Conclusion

This study demonstrated that the naturally derived temporin-SHf peptide is a potential antimicrobial and anticancer agent. Temporin-SHf is microbiocidal, non-hemolytic, and cytotoxic to human cancer cells but not to non-tumorigenic cells. It disturbs cancer cells' lysosomal integrity and causes cell membrane damage. The temporin-SHf inhibits A549 cancer cell proliferation and migration. It is anti-angiogenic and triggers caspase-dependent apoptosis through an intrinsic mitochondrial pathway. These findings provided strong evidence that the AMP database is a resource for developing new cancer therapeutics. The mechanism of action of temporin-SHf may help to understand the actual activity of AMPs in mammalian cells and provide potential theoretical support for future anticancer drug development. Also, it will help to provide accessible and cheap traditional medicine for cancer treatment.

## Experimental

### Chemicals

The important chemicals and reagents used in this study are mentioned with their CAS/catalog numbers in parentheses. Rink amide resin (183,599-10-2), Hexafluorophosphate Benzotriazole Tetramethyl Uronium (HBTU) (94,790-37-1), Reagent-K (TFA/H_2_O/Phenol/EDT/Thioanisole (85:5:5:2.5:2.5)), Acetonitrile (5-05-8), Trifluro acetic acid (TFA) (76-05-1), Dimethyl sulfoxide (DMSO) (67-68-5), Streptomycin (3810-74-0), Amphotericin-B (1397-89-3), MTT (thiazole blue tetrazolium bromide) (298-93-1), Neutral red (553-24-2), Lithium lactate (27,848-80-2), Nicotinotinamide adenine dinucleotide (NAD) sodium salt (20,111-18-6), Phenazine methosulfate (299-11-6), Iodonitrotetrazolium chloride (146-68-9), and Tween-20 (9005-64-5) were purchased from Merck Chemicals Co. USA. Tris (pH 8.0) (77-86-1), and SYBR Green qPCR master mix (4,309,155) was purchased from Thermo Fisher Scientific, USA. 5-Fluorouracil (5-FU) (51-21-8), Dulbecco's Modified Eagle's Medium (DMEM) (AT186), Fetal bovine serum (FBS) (9014-81-7), L-Glutamine (6-85-9), Sodium pyruvate (113-24-6), Penicillin (113-98-4), Human Umbilical Vein Endothelial Cells (HUVEC) (CL019), HEPES (4-(2-Hydroxyethyl) piperazine-1-ethane sulfonic acid) (7365-45-9), *N,N,N,N* tetramethyl ethylene diamine (TEMED) (110-18-9), Sodium lauryl sulphate (SDS) (151-21-3), Ethidium bromide (1239-45-8), Triton X-100 (9002-93-1) were purchased from HiMedia Laboratories Pvt. Ltd, Mumbai. Luria–Bertani (LB) broth (22,006), Potato dextrose agar (PDA) (71,788), Acetone (27,498), Ethyl acetate (141-78-6), Trichloro acetic acid (TCA) (76-03-9), Glucose (50-99-77), Ammonium bicarbonate (1066-33-7), Ether (60-29-7), Potassium phosphate (7778-77-0), Ethylene diamine tetra acetic acid (EDTA) (6381-92-6), Acetic acid (64-19-7), and n-butanol (71-36-3) were purchased from Sisco Research Laboratories (SRL), Mumbai, India. TRIzol reagent (ORN0102) was purchased from Origin, India.

### Selection of temporin-SHf

The topmost hydrophobic peptide, temporin-SHf, was selected based on its short length and broad-spectrum antimicrobial activity from publicly available antimicrobial peptide databases (http://aps.unmc.edu/AP/main.php, http://www.camp.bicnirrh.res.in, http://www.yadamp.unisa.it).

### Synthesis of temporin-SHf

**Fig. 19:**
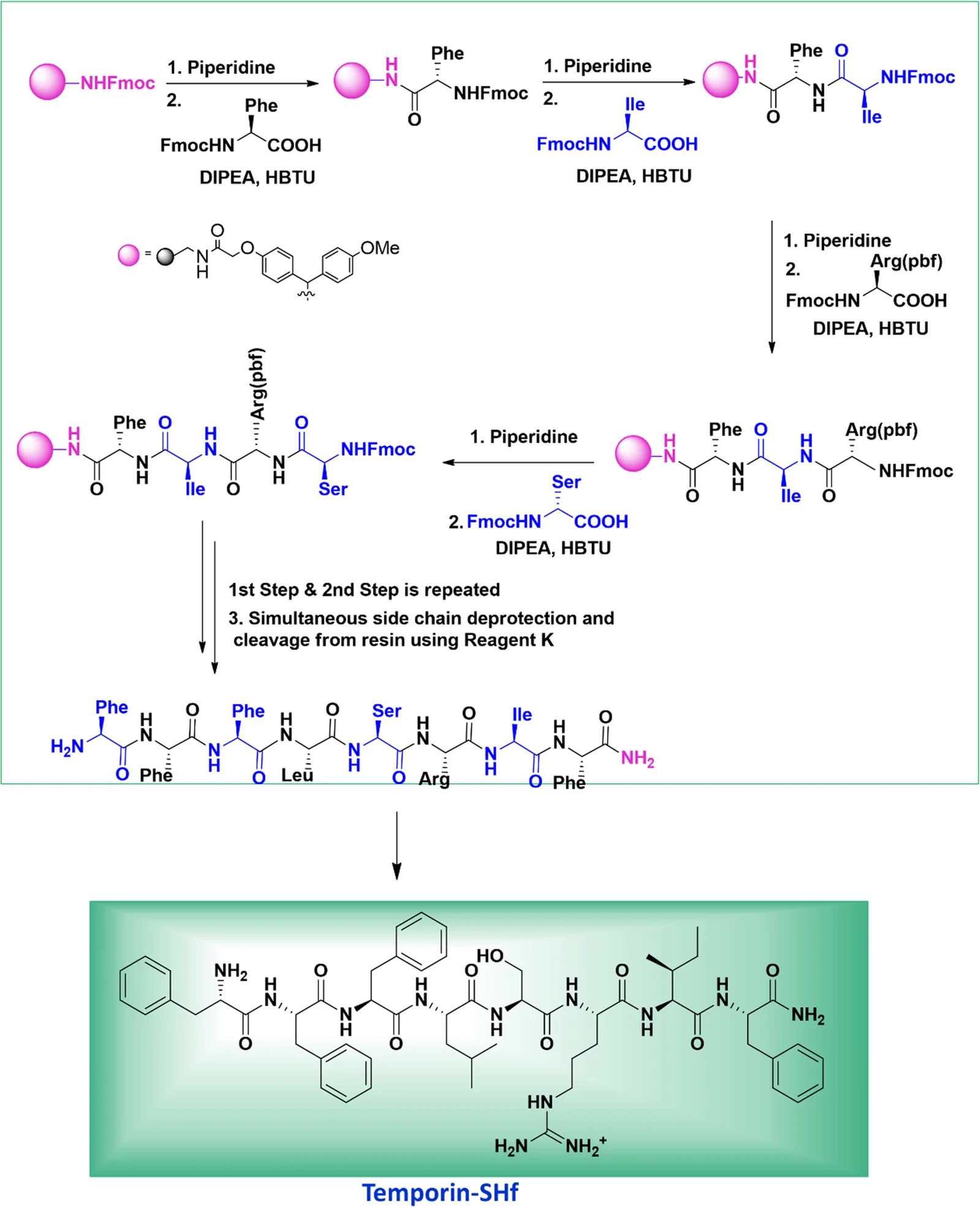
The synthesis of temporin-SHf by the solid-phase method

Temporin‐SHf was manually synthesized by standard solid phase peptide synthesis (SPPS) method using Fmoc (9-Fluorenylmethyloxycarbonyl) chemistry (Fig. 19). Rink amide resin of loading capacity 0.7 mmol was used as the solid support. The Fmoc-protected amino acids were coupled with the HBTU/DIPEA activation. Fmoc deprotection was carried out by using 20% piperidine in DMF solvent. After the successful coupling of amino acids, the peptide was cleaved off from resin using king's cocktail reagent TFA/H_2_O/Phenol/thionisole in the ratio of 85:5:5:2.5:2.5. The peptide was precipitated and washed 3–4 times with chilled ether. The crude peptide was washed 5–6 times with cold ether and purified by RP-HPLC on a semi-preparative Phenomenex C18-column (250 × 10 mm, 10 µM) over a linear gradient of acetonitrile (0.1% TFA). The flow rate was 2.0 mL/min, and elution was monitored at 226 nm.

### Analysis of physicochemical properties of temporin-SHf

Analyzed various physicochemical features to determine the peptide's antimicrobial and anticancer activities. The factors assessed were net positive charges, percent hydrophobic residues, hydrophobicity (H), and the grand average of hydropathicity (GRAVY), amphiphilicity (the mean hydrophobic moment), and secondary structure. The net charge and GRAVY of the amphibian skin peptide were computed using ProtParam (https://web.expasy.org/cgi-bin/protparam/protparam). Calculated the theoretical molecular mass of the peptide by peptide synthetics (http://www.peptidesynthetics.co.uk/tools/). The peptide hydrophobicity/hydrophilicity was analyzed using Peptide 2.0 Inc. (https://www.peptide2.com/N_peptide_hydrophobicity_hydrophilicity.php). We plotted the helical wheel of the peptide to predict their functional roles using Don Armstrong and Raphael Zidovetzki (https://pss.sjtu.edu.cn/cgi-bin/wheel.cgi; version ID: 0.10p06 12/14/2001 DLA). A helical wheel project revealed the distribution of cationic amino acids on the hydrophilic surface. Secondary structure predicted from amino acid sequences using PSIPRED (http://bioinf.cs.ucl.ac.uk/psipred/) and jpred4 (http://www.compbio.dundee.ac.uk/jpred4) prediction methods.

### Homology modeling analysis of temporin-SHf

The 3D structure of temporin-SHf was deduced using a homology modeling tool on the glide platform of Schrodinger software. Two related temporin peptides were available in PDB, namely, temporin-L (PDB ID-8TV4) and temporin-B (PDB ID-6GIL). The peptide temporin-L in SDS micelles was also available in the database (PDB ID-6GS5). The multiple sequence alignment of temporin peptides was achieved using Clustal omega. Due to close homology, temporin-L both in water and SDS micelles were used as templates to deduce the 3D structure of temporin-SHf in the corresponding medium. The input sequence of temporin-SHf was given in the structure prediction wizard of the homology modeling tool and subsequently modeled structures were built. Modeled structures were further processed using a protein preparation wizard that involves five steps-preprocess: review and modify, optimize, remove water, and minimization. The modeled structures were analyzed using the Ramachandran plot tool. The 3D structures were processed using the surface option of the style tool and distances were measured using the measure distance tool.

### Analysis of antimicrobial activities of temporin-SHf

#### Microbial strains and culture

Gram-positive bacteria: *Staphylococcus aureus* (MTCC 9542), *Bacillus subtilis* (ATCC 6051), *G*ram-negative bacteria: *Escherichia coli* (ATCC 25922), *Klebsiella pneumonia* (ATCC 13883), *Pseudomonas aeuginosa* (ATCC 15442) and *Aeromonas hydrophila* (ATCC 7966) strains were grown in Luria–Bertani (LB) broth at 37 °C in an incubator. *Aspergillus fumigatus* (ATCC 1022) and *A.niger* (ATCC 1015) fungi were grown in potato dextrose agar (PDA) at 25 °C in an incubator. These microbial strains were a generous gift from Dr. Buddolla's Institute of Life Sciences, Tirupati, India.

#### Minimal inhibitory concentration (MIC) of temporin-SHf for bacterial strains

Bacterial strains were grown in LB broth at the mid-log phase (OD_600_ = 0.6) and were made to 1 × 10^6^ cfu/mL. The stock concentration of temporin-SHf was prepared and diluted in LB broth to make the concentration range from 1 to 100 µM. 100 µL of each bacterial strain was added to 96-well plates along with 100 µL of different concentrations of peptide solution and incubated for 18 h at 37 °C in a shaking incubator. Phosphate-buffered saline (PBS) and streptomycin (10 µg) were used as negative and positive controls, respectively. Recorded absorbance at 600 nm using a microplate reader (EnSpire™ Multimode Plate Reader, PerkinElmer, Inc) to assess the bacterial growth. MIC value determines the minimum amount of peptide required to inhibit the visible growth of bacteria (Wiegand et al. 2008).

#### Temporin-SHf bacterial killing kinetic analysis

The killing kinetics of the temporin-SHf against *E.coli, S.aureus,* and *P.aeuginosa* were analyzed at their respective MIC concentrations. Bacterial strains in the mid logarithmic growth phase (OD_600_ = 0.6) were diluted to get 10^6^ cfu/mL and incubated with the temporin-SHf at different time intervals 20, 40, 60, 80, 100, 120, 140, 160 and 180 min. Aliquots drawn at these time points were plated on LB agar, incubated at 37 °C for 18 h, and counted colonies (Abraham et al. 2014).

#### Minimal fungicidal concentration (MFC) of temporin-SHf for fungal strains

The *A. fumigatus* and *A. niger* fungus were grown in potato dextrose (PD) medium for 24 h at 25 °C with an inoculum of 5 × 10^4^ cfu/mL. The stock concentration of temporin-SHf was prepared and diluted in a PD medium to make the concentration range from 1 to 500 µM. 100 µL of each fungal strain was added to 96-well plates and 100 µL of different concentrations of peptide solution. After incubation, absorbance was recorded at 600 nm using a microplate reader (EnSpire™ Multimode Plate Reader, PerkinElmer, Inc). PBS and amphotericin-B (20 mcg) were negative and positive controls (Contreras-Lynch et al. 2017).The scanning electron microscope (SEM) analysis was performed to validate the peptide is killing the fungi. The samples were dried and mounted on carbon tape placed on an aluminum stub. The samples were gold-coated by sputter deposition for 5 min. Samples were imaged in the Zeiss Field Emission Scanning Electron Microscope (FESEM) facility at DST-PURSE, Mangalore University, Mangalore, India.

#### Temporin-SHf fungi killing kinetic analysis

The killing kinetics of the temporin-SHf against *A. niger* and *A. fumigatus* were analyzed at their respective MFC concentrations. *A. niger* or *A. fumigatus* with an inoculum of 5 × 10^4^ cfu/mL were incubated with temporin-SHf at different time intervals 4, 8, 12, 16, 20, 24, 28, and 32 h. Aliquots drawn at these time points were plated on PDA and the colony number after incubating the plates at 28 °C for 24 h.

#### Hemolysis assay

Human peripheral blood (5 mL) was collected by venipuncture from a non-smoker, non-alcoholic, and medication-free healthy male and female volunteers of age between 20 and 23 years (21.11 ± 0.745) in vacutainer blood tubes containing sodium heparin (Cat. No. 367878, Becton–Dickinson, India Pvt. Ltd). The study was approved by the Central University of Kerala Institutional Human Ethical Committee (CUK/IHEC/2017-010). The volunteer's blood was drawn after the informed consent. The erythrocytes were isolated by the Ficoll-Paque density gradient method. Briefly, 5 mL of blood was diluted with 5 mL of sterile PBS, pH 7.4 (1:1 ratio). A total of 10 mL of diluted blood was layered on the 5 mL Ficoll-Paque-containing centrifuge tube. It was centrifuged at 400×*g* for 20 min at 20 °C to obtain a bottom layer containing erythrocytes. The bottom layered erythrocytes were collected and washed twice with PBS by centrifugation 800×*g* for 10 min. 100 µL of different concentrations of temporin-SHf (10–500 μM) were added with 100 µL of erythrocyte (*A*_sample_) and incubated at 37 °C for 30 min. The cells were centrifuged at 3000×*g* for 5 min and measured the supernatant absorbance at 595 nm. For negative and positive control, erythrocytes in PBS (*A*_blank_) and in 1% Triton X-100 (*A*_triton_) were used, respectively (Oddo and Hansen 2017). The percentage of hemolysis was calculated: [(*A*_sample_ − *A*_blank_)/(*A*_triton_ − A_blank_)] × 100.

### Antitumour activities of temporin-SHf

#### Cell lines and culture

Cancer cell lines MCF-7 (breast cancer), A549 (adenocarcinoma human alveolar basal epithelial cells), HepG2 (human liver cancer), and PC3 (prostate cancer) were collected as a gift from Prof. Paturu Kondaiah, Molecular Reproduction, and Developmental Genetics (MRDG), Indian Institute of Science (IISc), Bangalore, India. These cancer cells were cultured in Dulbecco's Modified Eagle's Medium (DMEM) containing 10% fetal bovine serum (FBS), 1% L glutamine, 1% sodium pyruvate, 50 U/mL penicillin, and 50 mg/mL streptomycin in a CO_2_ incubator at 37 °C and 5% CO_2_. Human Umbilical Vein Endothelial Cells (HUVEC) were cultured in an Endothelial Cell Expansion Medium (ECEM) containing reduced Serum w/VEGF, L-Glutamine, and Sodium bicarbonate w/o BBE at 37 °C in a controlled humidified environment with 5% CO_2_.

#### Cell viability and metabolic activity analysis

The primary cell viability evaluation method is the MTT (thiazole blue tetrazolium bromide) assay. MTT is a water-soluble tetrazolium salt. It converts to an insoluble purple formazan by cleaving the tetrazolium ring with the mitochondrial enzyme succinate dehydrogenase (complex II). The impermeable formazan accumulates inside the healthy metabolically active cells (Mosmann 1983). The cytotoxic nature of temporin-SHf on A549, MCF-7, HepG2, and PC3 human cancer cells and HUVEC, the primary non-tumorigenic cells, was determined by MTT assay. The cancer cells (10^5^ cells/mL) were seeded in 96-well sterile plates and treated with different temporin-SHf concentrations (10–100 µM). Simultaneously, PBS and 10% DMSO were treated as negative and positive controls for the seeded cells. The peptide, PBS, and DMSO-treated cells were incubated for 6, 12, 24, and 48 h at 37 °C in a controlled humidified environment with 5% CO_2_. After the respective incubation period, 10 µL of 5 mg/mL MTT solution was added to each well and then incubated for 4 h in dark conditions. The purple–blue MTT formazan formed depending on the cell viability, and it was dissolved in 150 µL of 100% DMSO. The absorbance was recorded using a microplate reader (EnSpire™ Multimode Plate Reader, PerkinElmer, Inc.) at 570 nm and 630 nm. The percentage of cell viability and inhibitory concentrations (IC_50_) were determined. The sub-lethal concentrations of 2.5, 5, and 10 µM temporin-SHf were selected for subsequent experiments. The IC_50_ values (Table 1) of the A549 cancer cells showed higher sensitivity to the temporin-SHf; hence, A549 cancer cells were chosen for all subsequence analyses.

#### Lysosomal integrity analysis

The neutral red uptake (NRU) assay determines the amount of neutral red dye entrapped in metabolically active cell lysosomes. There is a direct correlation between the ability of cells to preserve lysosomal integrity and the number of viable cells in culture (Repetto et al. 2008). A549 cells (10^5^cells/mL) were seeded in 96-well sterile plates and treated with different temporin-SHf concentrations. PBS and 10% DMSO-treated cells were negative and positive controls, respectively. The respective treated cells were incubated for 24 h at 37 °C with 5% CO_2_. After incubation, 10 µL of 1% neutral red solution was added to each well and then incubated in the dark for 3 h at 37 °C with 5% CO_2_. The cells were washed twice with PBS to remove the excess solution. 100 µL of desorbing reagent (1% glacial acetic acid and 50% ethanol) were added to each well to dissolve dye trapped in the cells and incubated for 30 min at 37 °C. The absorbance was recorded using a microplate reader (EnSpire™ Multimode Plate Reader, PerkinElmer, Inc.) at 540 nm. The viability (%): optical density [OD] of the treated group/OD of the control group*100. The viability of the control group was set to 100%.

#### Cell membrane integrity analysis

Temporin-SHf-caused cell death/necrosis was evaluated by releasing the cytoplasmic enzyme lactate dehydrogenase (LDH) into the culture medium (Kumar et al. 2018). Briefly, A549 cells (10^5^ cells/mL) were seeded in a 96-well plate for 24 h with or without temporin-SHf treatment. PBS and 10% DMSO-treated cells were negative and positive controls, respectively. After the incubation, the cell lysate was added to a solution mixture of 50 μL of 200 mM Tris (pH 8.0), 50 μL of 50 mM lithium lactate, and 50 μL of nicotinamide adenine dinucleotide sodium salt (NAD), phenazine methosulfate (PMS), iodonitrotetrazolium chloride (INT) and incubated for 5 min. The absorbance was recorded using a microplate reader (EnSpire™ Multimode Plate Reader, PerkinElmer, Inc.) at 450 nm and 680 nm. The viability (%) was expressed as (OD of the treated group/OD of the control group)*100. The viability of the control group was set to 100%.

#### Soft agar colony-forming assay

The effect of temporin-SHf on A549 cancer cell colony formation was analyzed using the method of Anjitha et al (2020) with minor modifications. Briefly, in a six-well plate, 0.6% agar bottom was layered. A549 (2 × 10^4^ cells/well) were added to the mixture of 0.3% agarose complete medium containing different concentrations of temporin-SHf (2.5, 5, 10 µM). PBS and 5 µM 5-Fluorouracil (5-FU) were negative and positive controls. Immediately, a 0.3% agar mixture was added on top of the 0.6% agar solid bottom layer and incubated at room temperature for 30 min to solidify. After polymerization, 100 µL of complete medium was added as a top layer. A feeder layer containing a mixture of agar and varying concentrations of temporin-SHf at 7-day intervals until colonies were visible (2.5 weeks). The controls and peptide-treated cells were cultured in a humidified incubator with 5% CO_2_ at 37 °C. An inverted microscope connected to a Magnus digital camera at 10× magnification was used to capture images and count colonies.

#### Tumor cell scratch assay

A549 cells were grown up to 90–95% confluency in a six-well plate. Then, the cell monolayer was scratched with a sterile 200 µL pipette tip and washed with a cell growth medium to remove the detached cells. It had a tunnel-like appearance in which both sides of the scratched area had cells. The cells were cultured in a serum-free medium and treated with or without different concentrations of temporin-SHf (2.5, 5.0, 10 µM) for 24 h (Hulkower and Herber 2011). An inverted microscope connected to a Magnus digital camera at 10X magnification was used to capture the monolayer's images at 0 h and 24 h. The % wound closure indicates the cancer cell migration. The images were analyzed using ImageJ software. Wound closure % = [0 h − 24 h/0 h] × 100.

0 h = the area of the wound measured immediately after scratching.

24 h = the wound area measured after the 24 h of the scratch.

#### Matrix metalloproteinases protein analysis by western blot method

A549 cells were seeded in 35 mm culture dishes at 10^5^ cells/mL density and incubated for 24 h. Then, cells were treated with or without temporin-SHf (2.5, 5, 10 µM) and incubated for 24 h. Subsequently, cells were washed twice with ice-cold PBS and lysed with RIPA lysis buffer (20 mM Tris–HCl buffer, pH 7.5, containing 1 mM EDTA, 1 mM DTT, 1 mM PMSF, and 1 mM protease inhibitor cocktail) for 30 min on ice. The debris was removed by centrifugation at 10,000×*g* for 20 min (Morita et al. 2009). The Bradford method (1976) determined the protein concentrations of supernatant. Equal protein (30 µg) samples were subjected to 12–15% SDS–polyacrylamide gel electrophoresis (PAGE) and the protein was transferred to a polyvinylidene difluoride (PVDF) membrane. The membrane was first incubated in a blocking solution (5% BSA) for 1 h and then incubated with primary antibodies matrix metalloproteinases (MMP-2 or MMP-9) (Cell Signaling Technology) for 2 h at room temperature. This was followed by washing with Tris-buffered saline with Tween-20 (TBST) (50 mM Tris HCl, 150 mM NaCl, 0.1% Tween-20, pH 7.5) three times and, subsequently, the membranes were incubated with HRP-labelled secondary IgG antibody (Cell Signaling Technology) for 1 h and then washed with TBST thrice. Finally, protein bands were visualized by an enhanced chemiluminescence (ECL) system (LI-COR, Inc, USA, Model-3600).

### Angiogenesis analysis

#### In vitro angiogenesis assay

An in vitro angiogenesis assay was conducted using the MILLIPORE kit manufacturer's instructions. First, a sterile microfuge tube added 100 µL of 10X diluent buffer to 900 µL of ECMatrix™ solution. Then, this solution was transferred (50 µL) to each well of a pre-cooled 96-well cell culture plate. The solution was solidified at 37 °C within 1 h. HUVEC were harvested and resuspended in endothelial cell expansion media (ECEM). 1 × 10^4^ cells were seeded per well onto the surface of polymerized ECMatrix™. These cells were cultured in a humidified incubator with 5% CO_2_ at 37 °C for 12 h. Then, cells were treated with/without temporin-SHf (2.5, 5.0, 10 µM). After 8 h of incubation, a cellular network was observed in control and temporin-SHf-treated cell culture plates. The network images were captured using an inverted microscope connected with a Magnus digital camera at 10× magnification. Analyzed the angiogenesis from the captured images using ImageJ software.

#### In vivo chicken embryo chorioallantoic membrane (CAM) assay

CAM proliferates and produces a rich vascular network, making it a convenient and versatile model for in vivo angiogenesis studies. The fertilized chicken (*Gallus gallus*) eggs were incubated in a humidified incubator at 37 °C and rotated once every 2 h. An air chamber was formed on the broad side of the egg (10 mm × 10 mm) small window was created by carefully removing the eggshell using sterile forceps on 72 h of incubation. Then, the clean filter paper discs soaked with/without temporin-SHf (2.5, 5, 10 µM) were placed on vascularized sites of the CAM. Finally, the window was covered using parafilm, and eggs were put back in the incubator at 37 °C with a relative humidity of 80% for 7 days (Ribatti 2017). At the end of the incubation period, eggs were opened, CAM was separated from chick embryos, fixed with 1 mL methanol: acetone fixing solution (1:1) for 15 min, and photographed. Using ImageJ software, the blood vessel density in the CAM was analyzed.

#### Angiogenesis gene expression analysis

The total RNA was isolated from the control and temporin-SHf-treated CAMs using the TRIzol reagent. RNA was quantified and normalized to 1 μg. 20 μL of cDNA was synthesized using a high-capacity Verso cDNA synthesis kit (Thermo Fisher Scientific). Quantitative real-time PCR (qRT-PCR) (Applied Biosystem; QuantstudioTM) analyzed the relative gene expression using the SYBR Green qPCR master mix. Actin served as the internal control. The chick *VEGF*, *VEGFR2*, and *FGF* gene primers were designed with Integrated DNA Technologies (IDT) (Table 2). Each sample was run in triplicate for each gene. The gene expression levels were analyzed by calculating ΔCT and ΔΔCT values: ΔCT = CT value of target gene–CT value of reference gene and ΔΔCT = ΔCT value of the test for target gene–ΔCT value of control for the target gene. The relative gene expression (RQ) is expressed as 2-ΔΔCT (Table 5).

**Table 5:** List of primers used for angiogenesis gene expression study

Gene	Forward primer	Reverse primer
*VEGF*	5′-GGAGTTGTCGAAGGCTGCT-3′	5′-TTGATAACTTCGTTGGGCTTC-3′
*FGF*	5′-TTCTTCCTGCGCATCAAC-3′	5′-CGATAGCTCGTCCAG-3′
*VEGFR2*	5′-GGGGAAGATGTACTCGGTGA-3′	5′-CATCCATGT TCAAACATCACAA-3′
*Actin*	5′-GCTCTGACTGACCGCGTT A-3′	5′-ACGAGCGCAGCAATATCAT-3′

### Apoptotic assays

#### Hoechst 33342 nuclear staining

Apoptotic cells were determined by staining with Hoechst 33342, a DNA-specific fluorescent dye, which stains the condensed chromatin of apoptotic cells more brightly than normal cells' chromatin. Morphological changes in the nuclear chromatin of cells undergoing apoptosis were observed with a fluorescent microscope (Leica, Germany) equipped with a UV filter. Briefly, temporin-SHf-treated and untreated A549 cells (10^5^ cells/mL) were cultured in a humidified incubator with 5% CO_2_ at 37 °C for 24 h. The respective experimental group cells were collected and washed with PBS. These cells were incubated with 10 μL/mL Hoechst 33342 for 15 min at 37 °C, then mounted on glass slides and observed under a fluorescent microscope at 20× magnification (Leica, Germany) (Crowley et al. 2016).

#### Acridine orange (AO)/ethidium bromide (EtBr) staining

A549 cells (10^5^ cells/ mL) were treated with/without temporin-SHf and were cultured in a humidified incubator with 5% CO_2_ at 37 °C for 24 h. The cells were washed with PBS and stained with 100 μg/mL AO and 100 μg/mL EtBr mixed in PBS in a 1:1 ratio (Kasibhatla et al. 2006). The cells were observed under a fluorescent microscope (Evos Thermo image station) at 20× using an excitation wavelength of 488 nm and an emission wavelength of 540 nm.

#### Apoptosis analysis by Annexin V and propidium iodide

For the identification of apoptotic cells via Annexin V and propidium iodide (PI), control and temporin-SHf-treated cells were detached by mild trypsinization, incubated with Add 100 µL 1X binding buffer and 5 µL Annexin V and 5 µL of Propium iodide (BD pharminogen, FITC Annexin V apoptosis detection kit). After 15 min incubation, flow cytometric analysis was performed with the CYFLOW space flow cytometry at the MAHE-Life sciences, Manipal.

#### Apoptosis protein expression analysis by western blot method

According to Morita et al (2009) and Bradford (1976), the proteins were isolated and estimated. All protocols were the same as in Matrix metalloproteinases protein analysis by western blot method**.** The primary antibodies phosphoinositide-3-kinase (PI3K), protein kinase B (Akt), Tumor suppressor protein P53 (p53), B-cell lymphoma 2 (Bcl-2), Bcl-2-Associated X Protein (Bax), Caspase 9/8/3, poly adenosine diphosphate-ribose polymerase (PARP), RIP and P-RIP (Cell Signaling Technology) were used to identify the apoptotic pathway.

#### Data analysis

All the experiments were conducted in triplicate, wherever applicable. The data obtained from the various experiments were presented as mean ± standard error of the mean (SEM). The data were analyzed by analysis of variance (ANOVA) followed by the Tukey HSD test's multiple comparisons. Adopted two levels of significance. Values are significant compared to control at *p < 0.05; **p < 0.01. Data not having any symbol are insignificant at *p* > 0.05. All the analyses were performed using SPSS (version 16.0) (SPSS Inc., Chicago, IL, USA).

## Supplementary Information

Below is the link to the electronic supplementary material.

Supplementary file1 (DOCX 14 KB)

Supplementary file2 (PPTX 781 KB)

## Data Availability

Data will be made available on request.
