# Supplementary material for: Antimicrobial and antitumor properties of anuran peptide temporin-SHf induce apoptosis in A549 lung cancer cells
Source: Amino Acids. 2024 Feb 6;56:12. doi: 10.1007/s00726-023-03373-3 (PMC10847208; doi:10.1007/s00726-023-03373-3)
Supplement: Supplementary file 1 [file 726_2023_3373_MOESM1_ESM.docx]

**Highlights**

- Temporin-SHf peptide is synthesized by solid-phase Fmoc chemistry.
- Temporin-SHf is an ultra-short, hydrophobic, cationic peptide.
- Temporin-SHf showed antimicrobial and antitumor activities.
- Temporin-SHf induces an intrinsic pathway of apoptosis in A549 cells.

**Fig. S1** RP-HPLC profile showing purity of the temporin-SHf peptide.

**Fig. S2.** Multiple sequence alignment of temporin-SHf with (a) temporin-L and (b) temporin-B.

**Fig. S3** (a) The Hydrogen bond network in the helical region of the modeled structure of temporin-SHf in micelles. (b) The aromatic Phe-Phe T-shaped interaction was observed in the modeled temporin-SHf in SDS micelles.
