# Supplementary material for: Antimicrobial and antitumor properties of anuran peptide temporin-SHf induce apoptosis in A549 lung cancer cells
Source: Amino Acids. 2024 Feb 6;56(1):12. doi: 10.1007/s00726-023-03373-3 (PMC10847208; doi:10.1007/s00726-023-03373-3)
Supplement: Supplementary file 2 — Supplementary file2 (PPTX 781 KB) [file 726_2023_3373_MOESM2_ESM.pptx]

## Slide 1
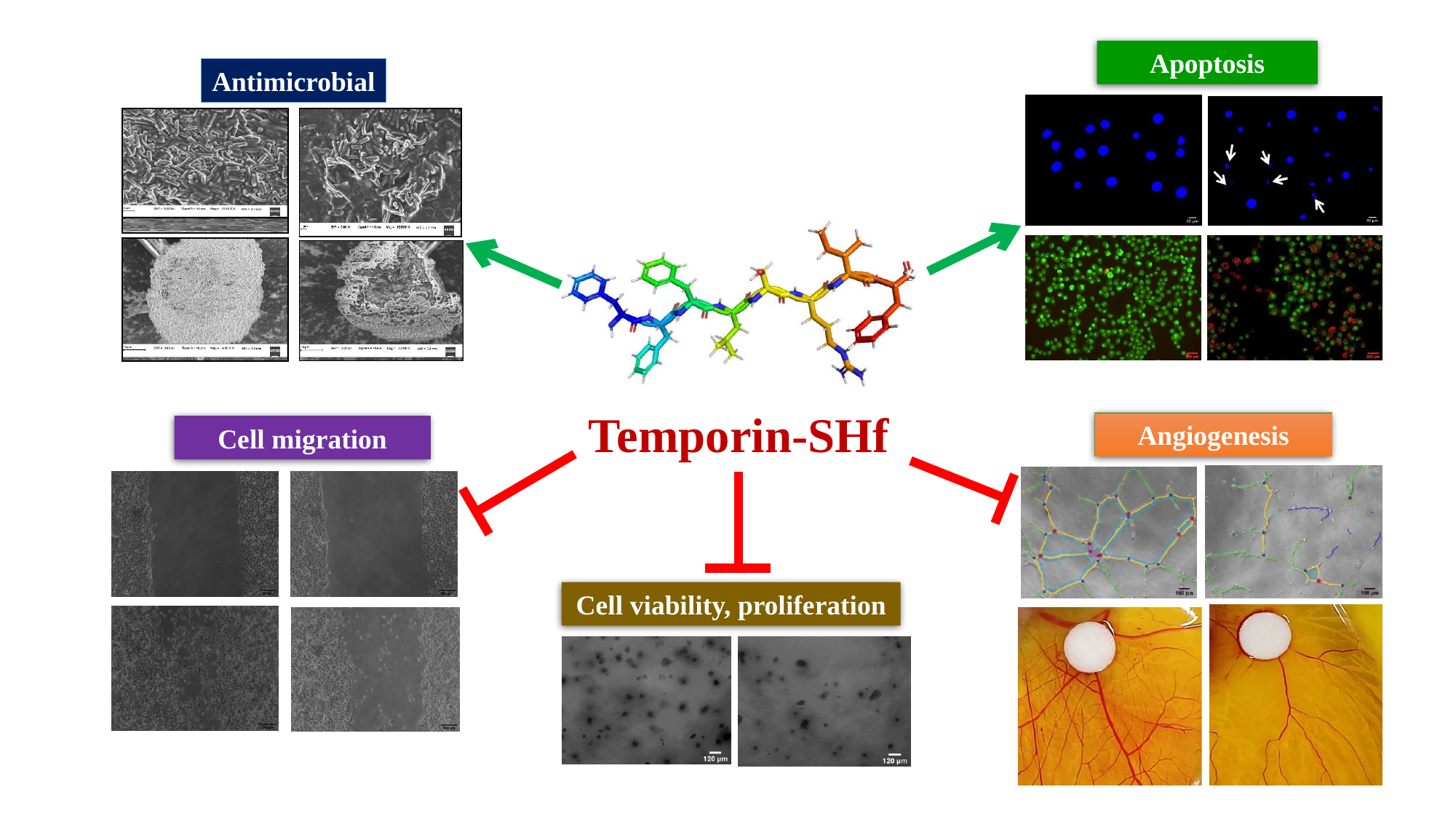

Apoptosis
Antimicrobial
Temporin-SHf
Angiogenesis
Cell migration
Cell viability, proliferation
